# Allosteric regulation of a prokaryotic small Ras-like GTPase contributes to cell polarity oscillations in bacterial motility

**DOI:** 10.1371/journal.pbio.3000459

**Published:** 2019-09-27

**Authors:** Jyoti Baranwal, Sébastien Lhospice, Manil Kanade, Sukanya Chakraborty, Priyanka Rajendra Gade, Shrikant Harne, Julien Herrou, Tâm Mignot, Pananghat Gayathri

**Affiliations:** 1 Indian Institute of Science Education and Research, Pune, India; 2 CNRS-Aix Marseille University, Laboratoire de Chimie Bactérienne, Marseille, France; Rutgers University-Robert Wood Johnson Medical School, UNITED STATES

## Abstract

Mutual gliding motility A (MglA), a small Ras-like GTPase; Mutual gliding motility B (MglB), its GTPase activating protein (GAP); and Required for Motility Response Regulator (RomR), a protein that contains a response regulator receiver domain, are major components of a GTPase-dependent biochemical oscillator that drives cell polarity reversals in the bacterium *Myxococcus xanthus*. We report the crystal structure of a complex of *M*. *xanthus* MglA and MglB, which reveals that the C-terminal helix (Ct-helix) from one protomer of the dimeric MglB binds to a pocket distal to the active site of MglA. MglB increases the GTPase activity of MglA by reorientation of key catalytic residues of MglA (a GAP function) combined with allosteric regulation of nucleotide exchange by the Ct-helix (a guanine nucleotide exchange factor [GEF] function). The dual GAP-GEF activities of MglB accelerate the rate of GTP hydrolysis over multiple enzymatic cycles. Consistent with its GAP and GEF activities, MglB interacts with MglA bound to either GTP or GDP. The regulation is essential for cell polarity, because deletion of the Ct-helix causes bipolar localization of MglA, MglB, and RomR, thereby causing reversal defects in *M*. *xanthus*. A bioinformatics analysis reveals the presence of Ct-helix in homologues of MglB in other bacterial phyla, suggestive of the prevalence of the allosteric mechanism among other prokaryotic small Ras-like GTPases.

## Abstract 2

A study on the mechanism of cell polarity oscillations in Myxococcus xanthus reveals a novel allosteric regulatory mechanism for a small Ras-like GTPase. The motility protein MglB is the first example of both GTPase activating protein (GAP) and guanosine nucleotide exchange factor (GEF) activities being integrated into a single regulator of the small Ras-like GTPase MglA.

## Introduction

**Fig 1:**
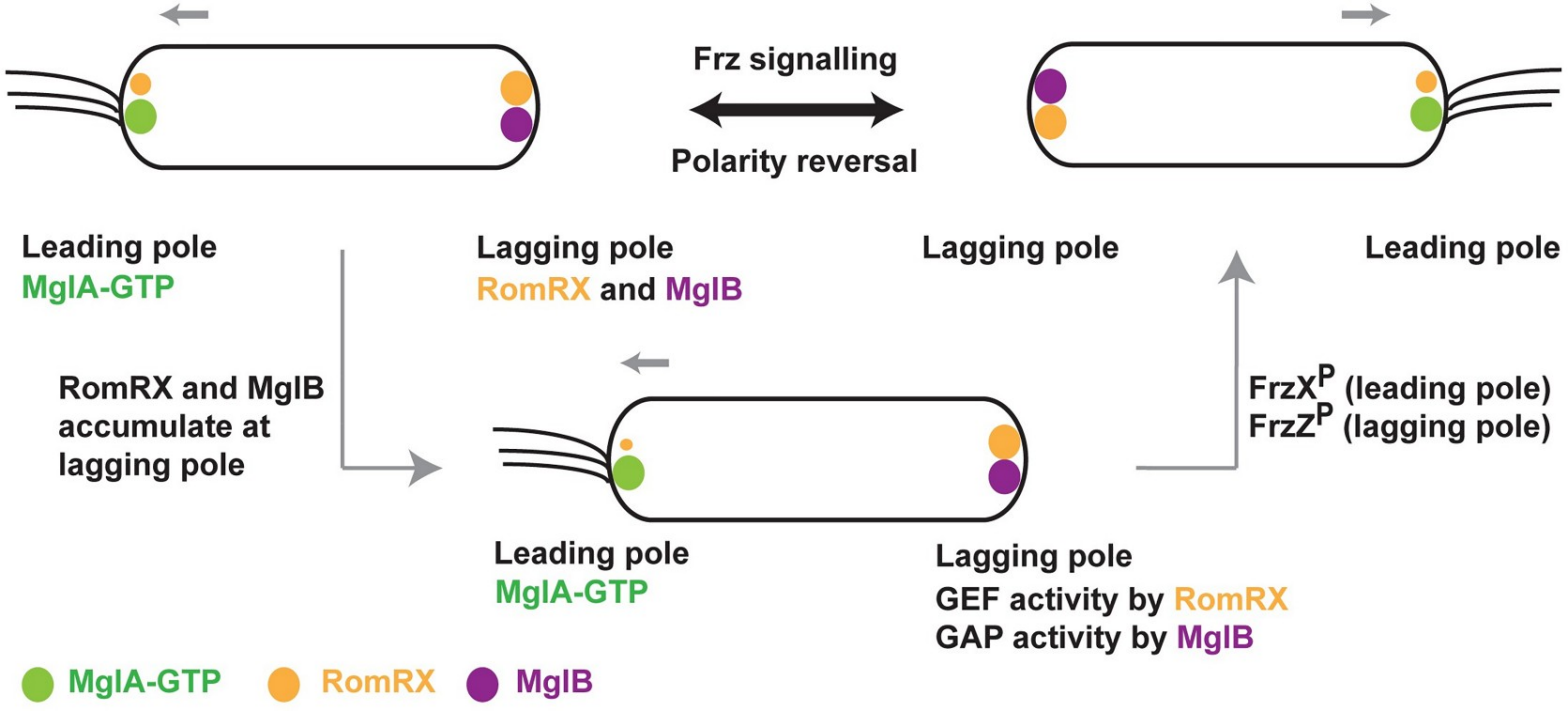
Localization of cell polarity determining proteins in *M*. *xanthus*. Schematic showing the localization of MglA, MglB, and RomRX in *M*. *xanthus*. MglA (GTP-bound state) localizes to the leading pole, whereas MglB and RomRX mainly localize at the lagging pole. A polarity reversal occurs when the proteins relocalize. A signaling cascade consisting of the Frz-signaling proteins drives the polarity reversals. MglA, MglB, and RomRX are represented as green, magenta, and orange spheres. The asymmetry in the size of orange spheres at the 2 poles denotes the asymmetric localization of RomR, with higher concentration at the lagging pole. FrzX^P^ and FrzZ^P^ denote phosphorylated states of FrzX and FrzZ, respectively. Frz, *frizzy*; Mgl, mutual gliding motility.

Small Ras-like GTPases are ubiquitous in eukaryotes and perform varied functions, including cell signaling and motility. Recently, such GTPases have been identified in prokaryotes too [1,2]. The soil bacterium *Myxococcus xanthus* exhibits 2 types of motility—adventurous gliding motility and social motility, which are essential for its normal life cycle [3]. The localization of motility complexes, and thereby the direction of movement, is determined by an oscillatory system, which includes Mutual gliding motility A (MglA), a small Ras-like GTPase, and Mutual gliding motility B (MglB), its GTPase activating protein (GAP) as major components [1,4,5]. Mutual gliding motility (*mgl*) was identified as a locus essential for both social and adventurous motilities in *M*. *xanthus*, comprising the 2 genes *mglA* and *mglB* [1,3]. Studies on MglA and MglB showed that these proteins exhibit a mutually exclusive localization pattern [4,6,7]. MglA localizes to the leading pole presumably in its GTP-bound state [4], whereas MglB localizes at the lagging pole (Fig 1). These studies [1,3–7] established the role of the prokaryotic small Ras-like GTPase in cell polarity determination and motility, which are functions analogous to its eukaryotic counterparts [8,9].

A remarkable feature of *Myxococcus* motility is the frequent reversals in its cell polarity in response to environmental cues [10]. *M*. *xanthus* changes its direction of movement by swapping the leading and lagging poles. The reversal frequency is regulated by the chemosensory-like frizzy (Frz) pathway, which relays environmental signals to the downstream MglA-MglB polarity control system [11–13] (Fig 1). High-resolution time-lapse experiments revealed that MglA and MglB relocalize sequentially during a reversal [4,7,11]. Importantly, the Frz system does not establish polarity, but its activity is required for inversion of the polarity axis. Another protein involved in the polarity control module is RomR, a protein with a response regulator receiver-like domain [14,15]. It was recently shown that RomR and its interacting partner RomX are essential for recruiting MglA-GTP to the poles [16]. Frz-signaling and RomR stimulates the pole-to-pole exchange of MglA and MglB [11,13–15]. All together, these proteins form a so-called gated relaxation oscillator, which functions to drive the polarity reversals in response to environmental signals [11].

Regulated reversals are centrally controlled by the MglA GTPase cycle. Though it is established that MglB functions as a GAP for MglA [4,7], proteins that function as guanine nucleotide exchange factors (GEFs) [17] for MglA were not identified until very recently [16]. The complex of RomR and RomX has been recently demonstrated to function as a GEF for MglA [16]. In vivo, the RomRX complex recruits MglA to the leading pole at the time of reversal. Between reversals, RomRX dissociates slowly and relocalizes to the lagging cell pole where RomR interacts with MglB [14,15]. Thus, both GAP and GEF activities accumulate at the lagging cell pole, preparing the next reversal event (Fig 1). However, at this stage, the cell will only reverse if signals from Frz are sufficiently strong. When this occurs, 2 response regulators FrzX^P^ (phosphorylated FrzX) and FrzZ^P^ (phosphorylated FrzZ) bind to opposite poles, which trigger the detachment of MglA from the leading cell pole and its relocalization to the lagging cell pole. MglB is subsequently detached and relocated to the opposite pole, completing the switch.

Many questions remain unaddressed concerning the exact sequence of events that take place at the poles. Specifically, the presence of antagonizing GAP and GEF activities at both cell poles requires that this balance be regulated. The accumulation of RomRX at the lagging pole is not sufficient to drag MglA to the lagging cell pole, most likely because the MglB GAP activity predominates. Accumulation of FrzX^P^ at the lagging pole could switch this balance in favor of the GEF and provoke the switch. In this study, we show that MglB is in fact a bifunctional enzyme and that MglB regulation could be key for the control of this enzymatic balance at the pole.

Previously, biochemical and structural studies with homologous proteins from *Thermus thermophilus* demonstrated that MglB is the GAP of MglA [4,5]. MglA and MglB from *T*. *thermophilus* (TtMglA and TtMglB) share 62% and 29% sequence identities, respectively, with the *M*. *xanthus* counterparts. Unlike the majority of small Ras-like GTPases, both the active site residues of the GTPase were found within TtMglA itself (Arg53^A^ and Gln82^A^; residue names with superscript “A” denote residues of MglA) instead of being contributed by the GAP [5]. A dimer of TtMglB interacts with a monomer of TtMglA. The structural studies revealed that β_2_ strand of TtMglA undergoes a screw-type movement during the interaction between TtMglA and TtMglB in the presence of GTP analogues [5]. The β-screw movement positions the active site residues favorably for GTP hydrolysis. In contrast to *M*. *xanthus*, *T*. *thermophilus* is not known to switch polarity and appears to lack most of the other regulatory components (e.g., Frz proteins). Hence, it is imperative to study the *Myxococcus* proteins for a complete molecular characterization of the oscillatory system.

In this study, we report the biochemical and structural characterization of *M*. *xanthus* MglA and MglB (MxMglA and MxMglB). Our investigations led to the discovery of an allosteric pocket on the small Ras-like GTPase fold. Allosteric binding of the C-terminal helix (Ct-helix) of MxMglB on MxMglA facilitates nucleotide exchange. Thus, MxMglB plays a dual role in increasing the GTPase activity of MxMglA by orienting the catalytic residues (role of a GAP) and by facilitating exchange of GDP with GTP (role of a GEF). Furthermore, our study reveals that the Ct-helix plays an important role in regulating the mutually exclusive localization of MglA, MglB, and RomR. Accordingly, deletion of the helix in *M*. *xanthus* trapped MglA, MglB, and RomR at both poles of the cell and drastically affected motility. Importantly, the study reveals the integration of GAP and GEF activities into a single interacting partner of a prokaryotic small Ras-like GTPase. Our study highlights the mechanistic features of a GEF in a prokaryotic small Ras-like GTPase family.

## Results

### Crystal structure of MxMglA is in the GDP-bound conformation

**Fig 2:**
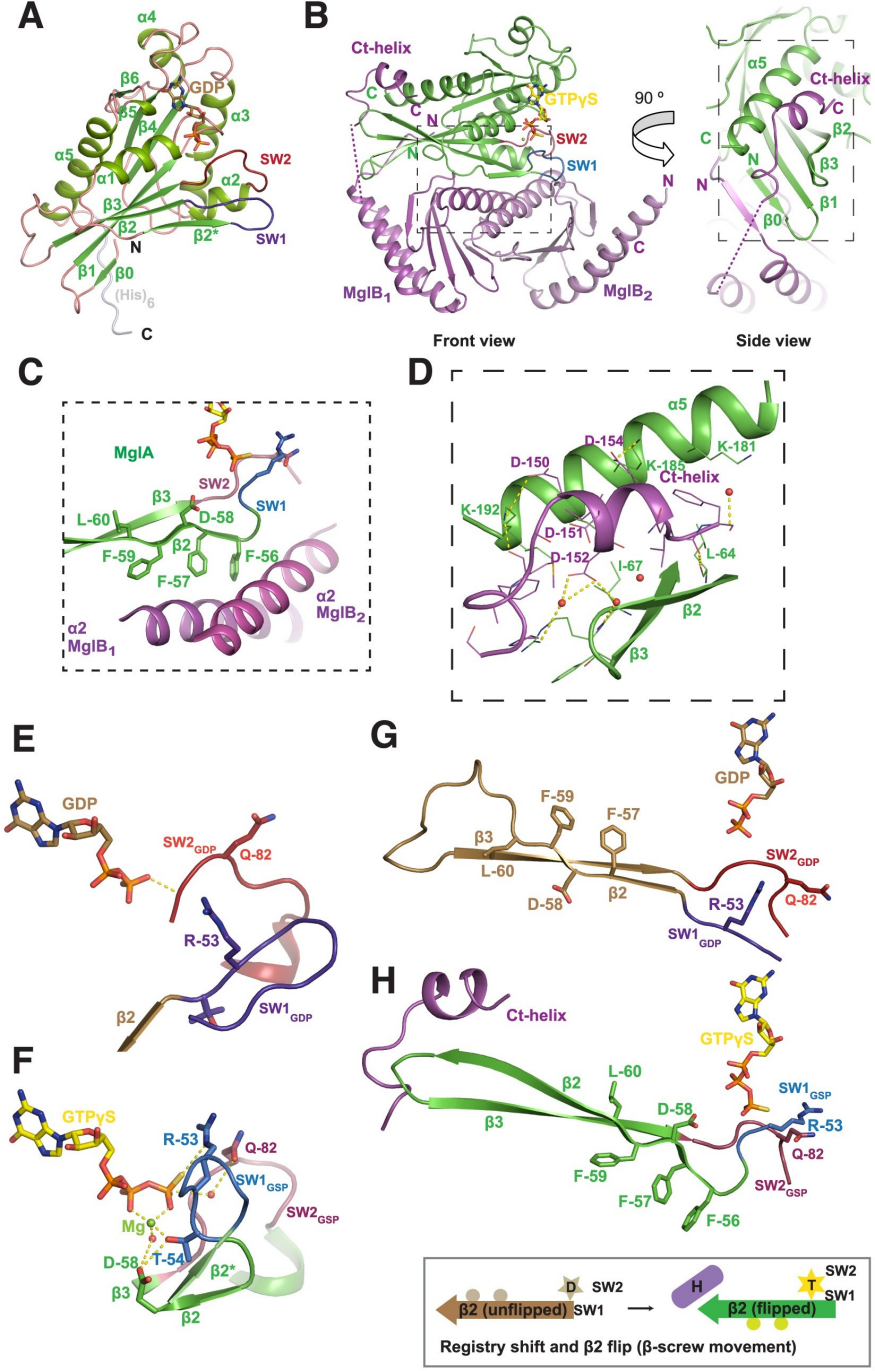
Crystal structures of *M*. *xanthus* MglA and MglAB–GTPγS complex. (A) Crystal structure of MxMglA bound to GDP. The secondary structure elements are labeled. GDP is shown in stick representation in light brown, and the catalytic loops switch 1 (blue) and switch 2 (red) are labeled as SW1 and SW2. The rest of the loops are shown in light pink, whereas the secondary structure elements are shown in shades of green. The C-terminal hexa-histidine tag (His)_6_, which is ordered in the crystal structure, is shown in gray. (B) Crystal structure of the complex of MxMglA (green) bound to GTPγS (yellow) and MxMglB (magenta). “Front” view and a 90° rotated view (side view) are shown. The 2 protomers of MxMglB are labeled as MglB_1_ and MglB_2_. The C-terminal helix of MxMglB (Ct-helix), the respective N- and C-terminal ends, switch 1 (blue; SW1) and switch 2 (red; SW2), and relevant secondary structures of MxMglA are labeled. GTPγS is shown in stick representation in yellow. Dotted magenta line connects the ends on either sides of the stretch of disordered residues in MglB_1_. Boxed regions in the front and side view panels highlight the major interfaces between MxMglA with the Rbl/LC7 domain of MxMglB (box with short dashes) and the Ct-helix of MxMglB (box with long dashes), respectively. (C) Residues of β_2_ strand (Phe56^A^, Phe57^A^, and Phe59^A^) of MxMglA (green) form a hydrophobic surface that interacts with MxMglB (magenta). The relevant secondary structure elements are labeled, and switch 1 and 2 (SW1 and SW2) are colored in blue and red, respectively. The region corresponds to a zoomed view of the boxed region (short dashes) in the front view of panel B. (D) The interacting interface of the Ct-helix of MxMglB (magenta) and MxMglA (green). The side chains of relevant interface residues are shown in stick representation and labeled. Water molecules are represented as red spheres, and dotted yellow lines represent hydrogen bond interactions. The region corresponds to a zoomed view of the boxed region (long dashes) in the side view of panel B. (E and F) Conformational changes in switch 1 (SW1; 2 shades of blue) and switch 2 (SW2; 2 shades of red) in the presence of MxMglB illustrates the mechanism of GAP activity (by reorientation of active site residues Arg53^A^ and Glu82^A^). MxMglA–GDP conformation and MglA from the MxMglAB–GTPγS complex are shown in panels E and F, respectively. The 2 structures from a superposed orientation are shown as separate panels. Secondary structures extending from the switch loops for MxMglA–GDP conformation and MglA from the MxMglAB–GTPγS complex are shown in brown and green, respectively. Switch 1 (or 2) of the 2 structures are labeled SW1 (or 2) and SW1 (or 2) using GDP and GSP as subscripts for the MxMglA–GDP and MxMglAB–GTPγS complexes, respectively. (G and H) Ct-helix of MxMglB (magenta) and GTP (represented by GTPγS shown in stick representation) bind to the C- and N-terminal ends of the β_2_ strand of MxMglA, respectively. MxMglA–GDP conformation and MglA from the MxMglAB–GTPγS complex are shown in panels G and H, respectively. The 2 structures from a superposed orientation are shown as separate panels. The unflipped and flipped states of the strand are shown (brown, MxMglA–GDP; green, MglA conformation in MxMglAB–GTPγS structure; switch 1 and switch 2 are in shades of blue and red, respectively). A schematic representation of the registry shift and β_2_ flip is shown below, with the same color scheme. A 6-pointed yellow star and a 5-pointed light brown star represent GTP (labeled as “T’) and GDP (labeled as “D”), respectively. The β_2_ strand is represented by an arrow. The residues that form the interface are schematically shown by 2 circles on the bottom or top of the β_2_ strand representation in the flipped and unflipped states of the strand, respectively. Ct-helix of MxMglB is schematically shown by a cylinder that is labeled “H.” Ct-helix, C-terminal helix; GAP, GTPase activating protein; GTPγS, guanosine 5’-O-[gamma-thio]triphosphate; Mgl, mutual gliding motility; MxMglA, *M*. *xanthus* MglA; MxMglB, *M*. *xanthus* MglB; Rbl, Roadblock; SW1, switch 1; SW2, switch 2.

The crystal structure of MxMglA was determined at a high resolution of 1.3 Å (Protein Data Bank [PDB] ID: 5YMX; S1 Table). Fig 2A contains a description of the small Ras-like GTPase fold of MglA with the secondary structure elements labeled. Structure of MxMglA is similar to TtMglA with 2 additional β-strands (β_0_ and β_2_*), compared with the 5 α-helices and 6 β-strands present in the small Ras-like GTPase fold [5]. All the residues of the small Ras-like GTPase fold as well as the C-terminal hexahistidine tag were modeled in the MxMglA structure. Though GDP was not added during protein purification or crystallization, the crystal structure of MxMglA contained GDP in the nucleotide-binding pocket (Fig 2A, S1A Fig). High-performance liquid chromatography (HPLC) analysis of the protein sample reconfirmed that the bound ligand is GDP (S1A Fig, S1B Fig).

### Structure of MxMglAB–GTPγS complex revealed a binding site for MxMglB Ct-helix

Crystal structure of the complex of MxMglA and MxMglB in the presence of guanosine 5’-O-[gamma-thio]triphosphate (GTPγS, a substrate analogue) (MxMglAB–GTPγS), was determined at a resolution of 2.4 Å (PDB ID: 6IZW; S1 Table). In the structure, a monomer of MxMglA was bound to a dimer of MxMglB (Fig 2B). MxMglA interacts with the MxMglB dimer through an extensive hydrophobic interface involving β_2_ strand of MxMglA and α_2_ helices of MxMglB monomers (Fig 2C), an interface similar to that of TtMglAB [5].

One of the novel features of the MxMglAB–GTPγS complex is the asymmetry between the 2 protomers of MxMglB (labeled MglB_1_ and MglB_2_; Fig 2B). MglB_1_ and MglB_2_ were asymmetric at their N- and C-terminal residues in the MxMglAB–GTPγS complex. The core of MxMglB, ranging from residues 10 to 131, belongs to the Roadblock/LC7 (Rbl/LC7) domain fold [18]. In MglB_1_, the N-terminal amino acids (2–7) comprised a β-strand that formed hydrogen bonds with the β_0_ strand of MxMglA, thus continuing the central β-sheet of the MxMglA fold (Fig 2B). α_1_ helix of the Rbl/LC7 fold started from Glu11^B^ (residue names with superscript B denote residues of MglB) of MglB_1_. In contrast, in MglB_2_, residues 1 to 7 were disordered, and the α_1_ helix comprised ordered residues starting from Tyr8^B^.

At the C terminus, density was absent for residues 131 to146. However, residues 147 to 157 of the MglB_1_ protomer could be modeled into the electron density (S1D Fig, S1E Fig), which adopted a helical conformation. Henceforth, this will be referred to as Ct-helix (C-terminal helix of MxMglB). In the MglB_2_ protomer, electron density was absent for residues beyond 130. The Ct-helix of MglB_1_ bound to a pocket in MxMglA distal from the nucleotide-binding site (Fig 2B and Fig 2D). The binding pocket in MxMglA was formed by the residues of the α_5_ helix, β_2_ strand, and the loop connecting β_2_ and β_3_ strands (β_2_–β_3_ loop) of MxMglA (interswitch region of small Ras-like GTPases [19]; Fig 2D). The corresponding β_2_ to β_3_ loop was disordered in the TtMglAB structure [5] (S1C Fig).

The conformational features described below are consistent with the previously determined structures of the corresponding complex from TtMglAB [5] (S1C Fig). Some of the prominent features are the optimal orientation of the active site residues (Fig 2E, Fig 2F) and the β-screw movement (Fig 2G, Fig 2H), which were also observed in the TtMglAB structures [5]. The conformational changes between MxMglA–GDP and the MxMglA–GTPγS bound to MxMglB resulted in movements of switch 1 and switch 2 such that Arg53^A^ and Gln82^A^ of MxMglA are now oriented towards the catalytic water (Fig 2E, Fig 2F). Another interesting observation is the orientation of the residues Thr54^A^ and Asp58^A^ of MxMglA, which coordinate the Mg^2+^ ion bound to GTPγS (Fig 2F). The β-screw movement exposed hydrophobic residues of MxMglA toward the MxMglB interface and facilitated MxMglAB interaction (Fig 2C, Fig 2H).

### Ct-helix allosterically affected the GTPase activity

**Fig 3:**
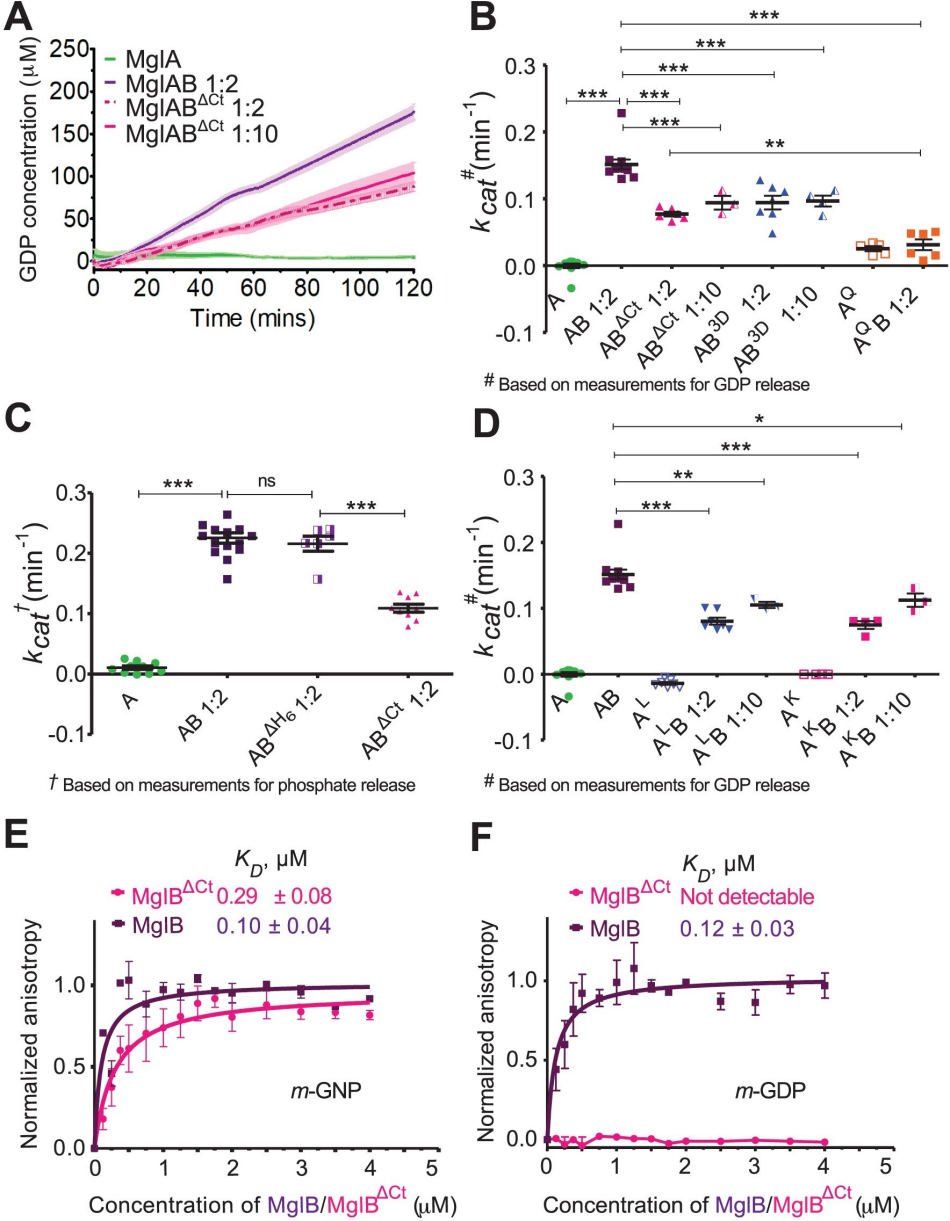
Ct-helix of MxMglB allosterically regulates GTPase activity of MxMglA. (A) GTPase activities of wild-type MxMglA only (green), and in the presence of MxMglB (dark purple; MglAB), and MxMglB^ΔCt^ (magenta; MglAB^ΔCt^). Ratio variations of 1:2 and 1:10 for MxMglB^ΔCt^ are shown in dotted and solid lines, respectively. The release of GDP was estimated using NADH-based enzyme-coupled assay. The lines represent the average of multiple repeats (at least 3), and the shaded zones represent standard error. (B) Comparison of *k*_*cat*_ values for MxMglA, MxMglAB, MxMglAB^ΔCt^, MxMglAB^3D^, and MxMglA^Q^B. The release of GDP was estimated using NADH-based enzyme-coupled assay. 1:2 and 1:10 represent the molar ratio of MxMglA and MxMglB monomers. (C) Comparison of *k*_*cat*_ values for MxMglA, MxMglAB, MxMglAB^ΔH6^ (MglB construct without a hexahistidine tag) and MxMglAB^ΔCt^. The release of phosphate was estimated using malachite green assay. 1:2 represents the molar ratio of MxMglA and MxMglB/MxMglB^ΔH6^/MxMglAB^ΔCt^ monomers. (D) Comparison of *k*_*cat*_ values for MxMglA, MxMglA^K^, and MxMglA^L^ in the presence of MxMglB. The release of GDP was estimated using NADH-based enzyme-coupled assay. 1:2 and 1:10 represent the molar ratios of MxMglA and MxMglB monomers. The data shown for MxMglA and MxMglAB have been duplicated from panel B for the sake of comparison. (E) Fluorescence anisotropy measurements for MxMglB (dark purple) and MxMglB^ΔCt^ (magenta) titrated against MxMglA–*m-*GNP, showing that both MxMglB and MxMglB^ΔCt^ bound to MxMglA in the presence of *m-*GNP. (F) Fluorescence anisotropy measurements for MxMglB (dark purple) and MxMglB^ΔCt^ (magenta) titrated against MxMglA–*m-*GDP, showing that MxMglB, but not MxMglB^ΔCt^, bound to MxMglA in the presence of *m-*GDP. The mean and 95% confidence intervals (long and short black horizontal lines, respectively) are shown for each sample in panels B, C and D. *p = 0.01–0.05, **p = 0.001–0.01, and ***p < 0.001. The numerical data for all the figure panels have been provided in the respective sheets in S1 Data. Ct-helix, C-terminal helix; *m-*GNP, 2’/3’-O-(N-Methyl-anthraniloyl)-guanosine-5’-[(β,γ)-imido]triphosphate; MxMglAB^ΔH6^, Mx MglA and MglB (without hexahistidine tag) complex; MxMglAB^3D^, Mx MglA and MglB D150A, D151A and D152A triple mutant); MxMglA^Q^B, MxMglA Q82L mutant; MxMglA, *M*. *xanthus* MglA; MxMglA^K^, MxMglA K181A and K185A double mutant; MxMglA^L^, MxMglA L64A and I67A mutant; MxMglB, *M*. *xanthus* MglB; MxMglB^ΔCt^, MxMglB Ct-helix truncation; ns, non-significant.

To elucidate the functional role of the MglB_1_ Ct-helix, we compared features of GTPase stimulation and MxMglA interaction with full-length MxMglB (MglB) and a truncated version of MxMglB lacking the Ct-helix (MglB^ΔCt^; S1F Fig). The GTPase activity was monitored by estimation of released GDP using a coupled enzyme assay [20] or phosphate release using malachite green–based assay [21]. The reactions were carried out with excess GTP (substrate) and hence measured multiple turnovers of the GTPase. Both approaches for monitoring enzyme activity showed similar rates of hydrolysis. The GTPase activity of MxMglA on its own was negligibly low, whereas the activity increased in the presence of MxMglB (Fig 3A, Fig 3B, Fig 3C, S2A Fig). This confirmed the role of MglB as a GAP, consistent with published results [4,5,7]. Control experiments were performed with only MxMglB (no MxMglA) and excess of MxMglB (S2A Fig). Similar GTPase activities were observed for MxMglA in the presence of MxMglB with or without a C-terminal hexahistidine tag (Fig 3C), and hence all further experiments were performed using the construct with C-terminal hexahistidine tag.

We found that the GTP hydrolysis rate was approximately 2-fold lower in the presence of MxMglB^ΔCt^ compared with MxMglB (Fig 3A, Fig 3B, Fig 3C). The GTPase activity remained low despite using 5 times excess of MxMglB^ΔCt^ (Fig 3A, Fig 3B), suggesting that the effect was not due to a reduced affinity of MxMglB^ΔCt^ to MxMglA. We compared the GTPase activities with that of the active site mutant MxMglA^Q82L^ (referred to as MxMglA^Q^) in the presence of MxMglB (S2B Fig, S2C Fig; Fig 3B). The GTPase activity of MxMglAB^ΔCt^ was, however, higher than that of MxMglA^Q^B.

Next, we proceeded to confirm that the interactions observed in the crystal structure between Ct-helix and the binding pocket of MxMglA are indeed relevant for the observed increase in GTPase activity. Analysis of the crystal structure of MxMglAB–GTPγS complex showed that Asp150^B^, Asp151^B^, and Asp152^B^ on the MxMglB Ct-helix formed water-mediated as well as direct interactions with MxMglA (Fig 2D). Lys181^A^ and Lys185^A^ of the α_5_ helix of MxMglA interacted with Phe157^B^ and Asp154^B^ of the MxMglB Ct-helix, respectively (Fig 2D). Leu64^A^ and Ile67^A^ of MxMglA were on the β_2_ to β_3_ loop (interswitch region) and were within interacting distances from the MxMglB Ct-helix (Fig 2D). There was a reduction in GTPase activity in the presence of MxMglB^3D^ (Fig 3B), a mutant of MxMglB in which Asp150^B^, Asp151^B^, and Asp152^B^ on the Ct-helix were replaced with alanine. Additionally, we studied the GTPase activities of the MxMglA mutants MxMglA^K^ (a double mutant of K181A and K185A) and MxMglA^L^ (a double mutant of L64A and I67A; S2B Fig, S2D–S2F Fig).

MxMglB-stimulated GTPase activity of the MxMglA mutants was less than the wild type (Fig 3D). The GTPase activity remained lower despite 5-fold increase in the concentration of MxMglB (Fig 3D). The above data demonstrated that mutation of residues in the helix-binding pocket affected rate of nucleotide hydrolysis, even though they were located away from the GTPase active site. This implied that MxMglB Ct-helix played an allosteric role in regulating the GTPase.

### Ct-helix facilitated interaction between MxMglB and GDP-bound MxMglA

Binding affinities of MxMglA with MxMglB and MxMglB^ΔCt^ in the presence of GDP and GTP, respectively, were estimated using fluorescence anisotropy measurements of *mant*-labeled nucleotides (fluorescent nucleotide analogues *m-*GDP or *m-*GppNHp, referred to as *m-*GNP hereafter) bound to MxMglA. Binding studies showed that *m-*GNP-bound MxMglA (MxMglA–*m-*GNP) bound to both MxMglB and MxMglB^ΔCt^, respectively (Fig 3E, S2A Table). The binding affinities of MxMglB to MxMglA^K^ and MxMglA^L^ in presence of *m*-GNP were also not affected drastically despite the mutations (S2F Fig, S2B Table). Hence, we concluded that the lower GTPase activities observed for these mutant constructs were not due to decreased affinity between the GTPase and MxMglB constructs.

Interestingly, MxMglB interacted with MxMglA in presence of either *m-*GNP or *m-*GDP (Fig 3E, Fig 3F). However, MxMglB^ΔCt^ bound to MxMglA in the presence of only *m-*GNP but not *m-*GDP (Fig 3E, Fig 3F). The binding affinities (reported as *K*_*D*_ values) between MxMglB and *m-*GNP–and *m-*GDP–bound MxMglA (0.10 ± 0.04 μM and 0.12 ± 0.03 μM, respectively) were comparable to the binding affinity of 0.29 ± 0.08 μM between MxMglB^ΔCt^ and *m-*GNP-bound MxMglA (Fig 3F, S2A Table). *K*_*D*_ between MxMglB^ΔCt^ and *m-*GDP-bound MxMglA could not be estimated, because the binding was insignificant (Fig 3F, S2A Table).

Thus, the Ct-helix appears to be essential for stabilizing the interaction between MxMglB and GDP-bound MxMglA. In the previous study with TtMglB, the binding between TtMglB and TtMglA was observed only in the presence of GNP (or GTP analogues), a characteristic feature of a GAP. Our studies imply that nucleotide hydrolysis by MxMglA is not sufficient for disrupting the MxMglAB complex in *M*. *xanthus*. Also, interaction with the GDP-bound state of a GTPase is a characteristic feature of a GEF [17]. Because we observed that MxMglA and MxMglB interact in the presence of GDP, we proceeded to check whether MxMglB could also perform a GEF function.

### Ct-helix of MxMglB contributed toward nucleotide exchange

**Fig 4:**
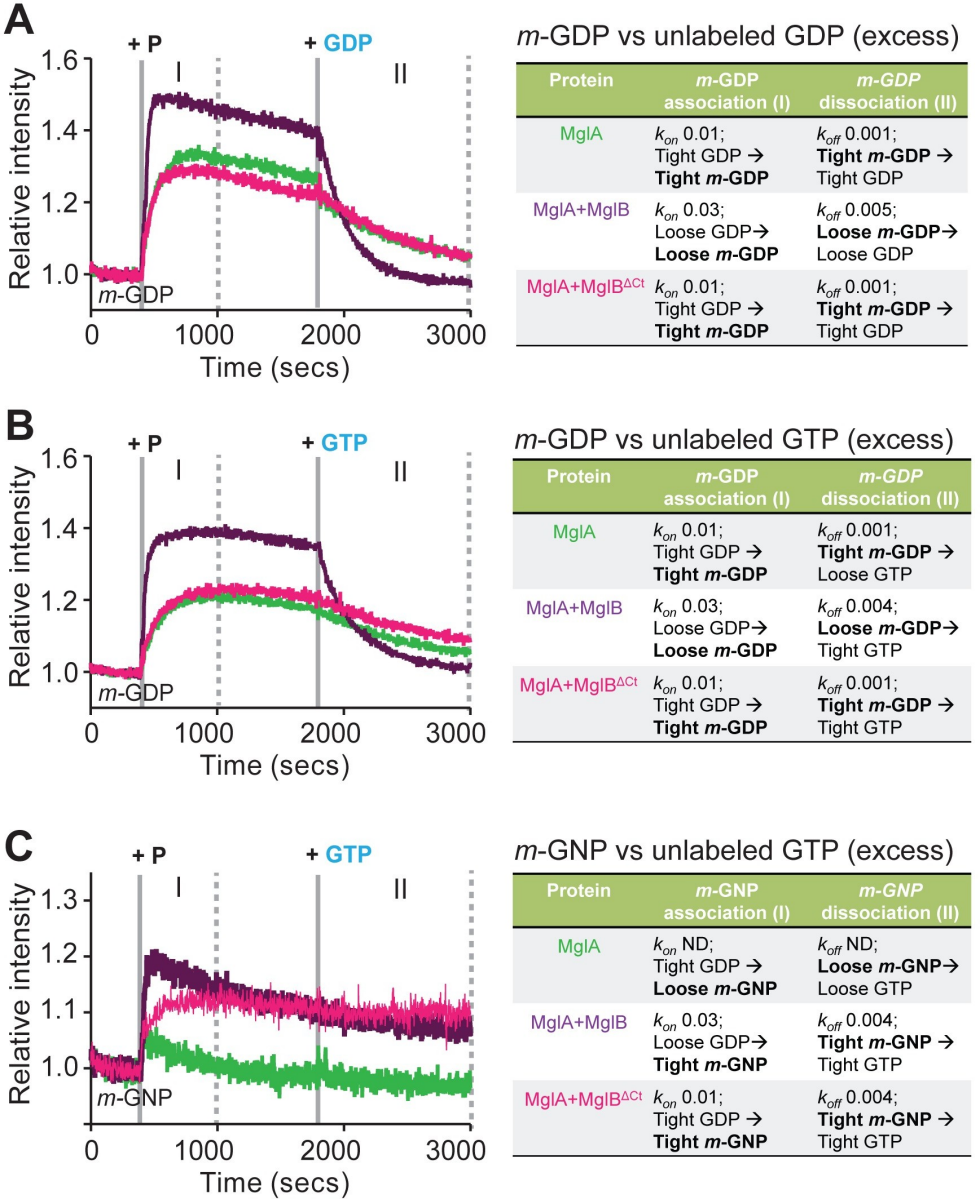
MglB Ct-helix facilitates nucleotide exchange of MglA. (A) Kinetic measurements of increase in *m-*GDP fluorescence (phase I) upon addition of MxMglA (green), mix of MxMglA and MxMglB in 1:2 ratio (dark purple), mix of MxMglA and MxMglB^ΔCt^ in 1:2 ratio (magenta) at 400 seconds (marked by solid grey line labeled “+ P”), followed by competition of *m-*GDP (phase II) by addition of excess of unlabeled GDP at 1,800 seconds (marked by solid gray line labeled “+ GDP”). (B) Kinetic measurements of increase in *m-*GDP fluorescence (phase I) upon addition of MxMglA (green), mix of MxMglA and MxMglB in 1:2 ratio (dark purple), mix of MxMglA and MxMglB^ΔCt^ in 1:2 ratio (magenta) at 400 seconds (marked by solid gray line and labeled “+ P”), followed by competition of *m-*GDP (phase II) by addition of excess of unlabeled GTP at 1,800 seconds (marked by solid gray line labeled “+ GTP”). (C) Kinetic measurements of increase in *m-*GNP fluorescence (phase I) upon addition of MxMglA (green), mix of MxMglA and MxMglB in 1:2 ratio (purple), mix of MxMglA and MxMglB^ΔCt^ in 1:2 ratio (magenta) at 400 seconds (marked by solid grey line labeled “+ P”), followed by competition of *m-*GNP (phase II) by addition of excess of unlabeled GTP at 1,800 seconds (marked by solid gray line labeled “+ GTP”). ND for the *k*_*on*_ and *k*_*off*_ values in panel C stands for “not determined”. For panels A to C, phase I (between solid gray line labeled “+ P” and the first dotted line) represents kinetics for association (for *k*_*on*_ estimation), phase II (between solid line labeled “+ GDP/GTP” and final dotted line) represents kinetics for dissociation (for *k*_*off*_ estimation). A schematic representation of the observations and probable explanations at each phase is shown on the right side, following the same color scheme for the font as in the plots. The events directly observable through fluorescence intensity change of the *mant*-labeled component in the experiment are highlighted in bold. The panels include representative plots of multiple repeats, and the *k*_*on*_ and *k*_*off*_ values (in units of s^-1^) represent the average values of the multiple repeats. The numerical data for all the figure panels have been provided in the respective sheets in S1 Data. Ct-helix, C-terminal helix; *m-*GDP, 2’/3’-O-(N-Methyl-anthraniloyl)-guanosine-diphosphate; MxMglA, *M*. *xanthus* MglA; *m-*GNP, 2’/3’-O-(N-Methyl-anthraniloyl)-guanosine-5’-[(β,γ)-imido]triphosphate; MxMglB, *M*. *xanthus* MglB; MxMglB^ΔCt^, *M*. *xanthus* MglB with Ct-helix truncated; ND, not determined.

As mentioned earlier, the purified MxMglA contained GDP bound to the active site pocket (S1 Fig). To observe the loading of *m*-GDP to MxMglA, fluorescence intensity of *m*-GDP was monitored upon addition of MxMglA. An increase in fluorescence intensity indicated increased binding of *m*-GDP. Exchange was observed to occur spontaneously upon addition of MxMglA but was accelerated upon addition of a mix of MxMglA and MxMglB (Fig 4A, phase I). In contrast, the presence of MxMglB^ΔCt^ instead of MxMglB did not affect the exchange kinetics (Fig 4A, phase I). The trend did not change despite addition of MxMglB^ΔCt^ in a 1:10 ratio (S2G Fig). Next, we monitored the exchange kinetics by competing the bound *m*-GDP with excess of unlabeled GDP. The rate of loss of fluorescence (*k*_*off*_) was higher for MxMglA+MxMglB mix compared with MxMglA or MxMglA+MxMglB^ΔCt^ mix (Fig 4A, phase II). This suggested that full-length MxMglB assisted in GDP exchange, whereas the C-terminal deletion mutant failed to stimulate GDP exchange.

We proceeded to carry out the same experiment by competing *m*-GDP with GTP, a relevant reaction for a GEF activity. MxMglB addition affected the rate of displacement of *m*-GDP in this experiment too (Fig 4B; phase II). Loading with *m*-GNP and competition with unlabeled GTP provided further information on the exchange kinetics (Fig 4C). Lack of a major increase in fluorescence indicated that binding of *m*-GNP to MxMglA was very unstable (Fig 4C, phase I). This, presumably, is due to the presence of unlabeled GDP prebound to the MxMglA sample. However, addition of either MxMglB or MxMglB^ΔCt^ with MxMglA stabilized the binding of *m*-GNP (Fig 4C, phase I). The rate of exchange of prebound GDP (from purification of MxMglA) with *m-*GNP (*k*_*on*_; Fig 4C, phase I) for MxMglA+MxMglB mix was 3 times faster than that of MxMglA+MxMglB^ΔCt^ mix. Subsequent competition of *m*-GNP with unlabeled GTP in the MxMglA+MxMglB mix and the MxMglA+MxMglB^ΔCt^ mix showed very slow or negligible exchange (Fig 4C, phase II).

These experiments suggested that MxMglA favored binding of GDP over GTP. However, in the presence of MxMglB, GTP bound better than GDP, as evidenced by a higher *k*_*on*_ and higher *k*_*off*_ for *m*-GDP and a higher *k*_*on*_ but a lower *k*_*off*_ for *m*-GNP (Fig 4C). In contrast, MxMglB^ΔCt^ did not affect the MxMglA–GDP complex (equal *k*_*on*_ for *m*-GDP for MxMglA and MxMglA+MxMglB^ΔCt^ mix; Fig 4A, phase I). However, it stabilized the GTP-bound conformation of MxMglA (increased intensity for *m*-GNP exchange; Fig 4C, phase I). This effect is consistent with our observations that MxMglB interacts with both MxMglA–GDP and MxMglA–GTP, whereas MxMglB^ΔCt^ interacts only with MxMglA–GTP (Fig 3E, Fig 3F). Thus, MxMglB functions not only by stabilizing GTP-bound MxMglA but also by destabilizing the bound GDP, whereas MxMglB^ΔCt^ contributes only toward stabilizing GTP-bound MxMglA.

Our assays measuring exchange kinetics with different combinations of labeled and unlabeled nucleotide pairs clearly implied that the Ct-helix played a role in nucleotide exchange. Consequently, we concluded that MxMglB accelerates MxMglA GTPase not only by appropriately orienting the catalytic residues but also by facilitating GDP release through an allosteric effect via the Ct-helix. This effect increased the overall hydrolysis rate in our GTPase assays, which are multiple turnover enzymatic reactions.

### *mglB*^ΔCt^ does not respond to frz signaling similar to Δ*mglB*

**Fig 5:**
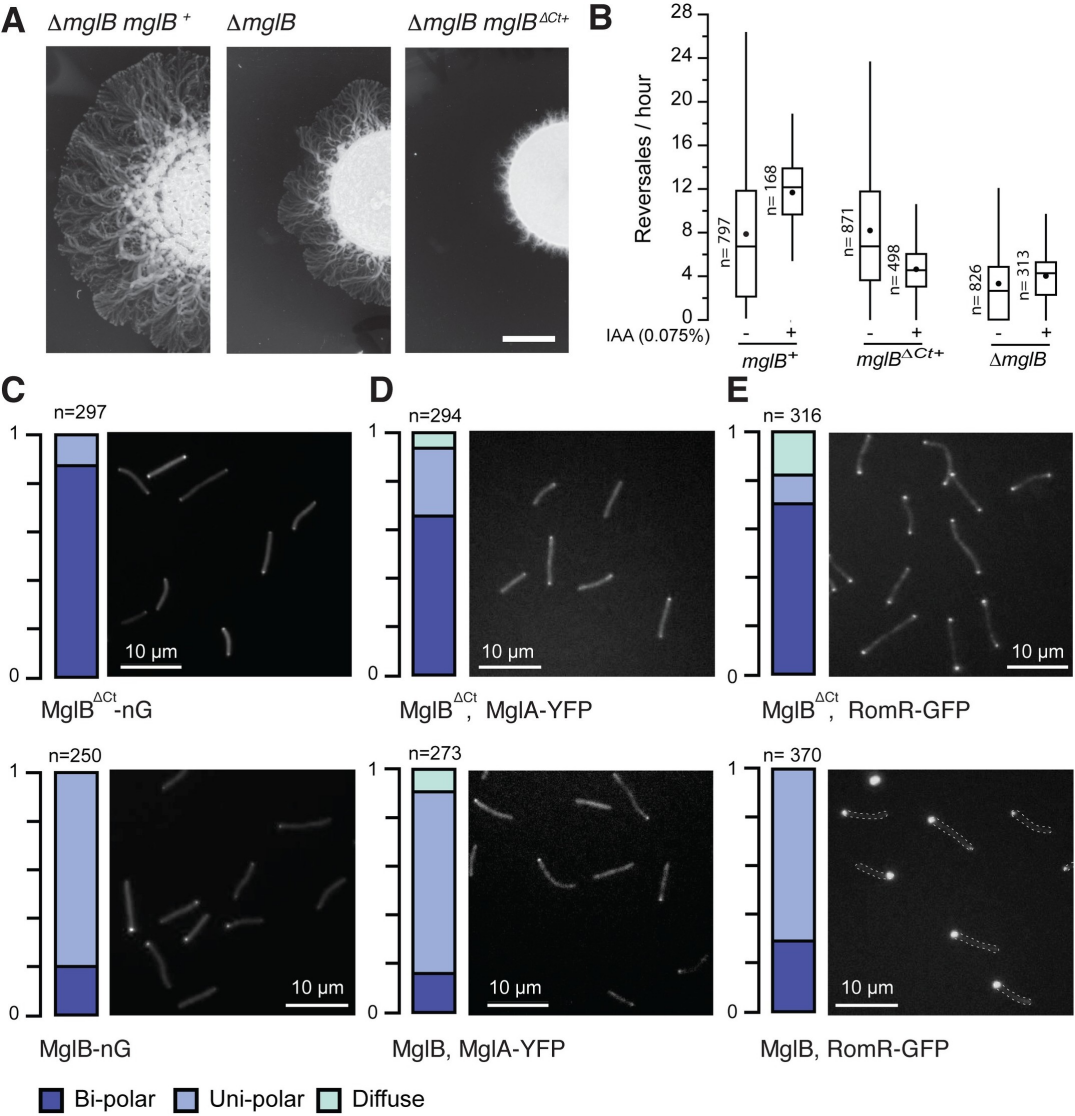
MxMglB Ct-helix is required for MxMglB function in vivo. (A) Expression of MxMglB^ΔCt^ leads to motility defects. Motility on 0.5% agar is shown after 48 hours of incubation at 32°C. Scale bar = 1 cm. (B) Reversal frequency of *M*. *xanthus* cells of Δ*mglB* mutant strain and cells expressing wild-type MxMglB (*mglB*^*+*^) or MxMglB^ΔCt^ (*mglB*^Δ*Ct+*^). The reversal frequencies were scored in absence (−) or presence (+) of 0.075% IAA, a condition known to activate the Frz system. For each strain and condition, the number of trajectories analyzed (*n*), the median (vertical line), and the mean (black dot) are reported for each box-and-whisker plot. (C) MxMglB^ΔCt^ shows a bipolar localization pattern. Representative fields of cells expressing MxMglB^ΔCt^-nG and MxMglB-nG are shown in top and bottom images, respectively. The fractions of cells exhibiting bipolar (dark blue), unipolar (light blue), and diffuse (cyan) localization are depicted on the left for each of the field views, in which “*n*” represents the total number of cells analyzed. The fluorescence intensity profiles for each cell are compiled and shown in S4A Fig. (D) MxMglA shows a bipolar localization pattern in the presence of MxMglB^ΔCt^. Representative fields of cells expressing MxMglA-YFP in *mglB*^Δ*Ct+*^ and *mglB*^+^ strains are shown in top and bottom images, respectively. The fractions of cells exhibiting bipolar, unipolar, and diffuse localization are depicted on the left for each of the field views, in which “*n*” represents the total number of cells analyzed. The fluorescence intensity profiles for each cell are compiled and shown in S4B Fig. (E) RomR shows a bipolar localization pattern in the presence of MxMglB^ΔCt^. Representative fields of cells expressing RomR-GFP in *mglB*^Δ*Ct+*^ and *mglB*^+^ strains are shown in top and bottom images, respectively. The fractions of cells exhibiting bipolar, unipolar, and diffuse localization are depicted on the left for each of the field views, in which “*n*” represents the total number of cells analyzed. In the bottom panel (RomR-GFP, *mglB*^+^), dashed lines indicate bacterial contour. The fluorescence intensity profiles for each cell are compiled and shown in S4B Fig. The numerical data for all the figure panels have been provided in the respective sheets in S1 Data. Ct-helix, C-terminal helix; Frz, frizzy; IAA, isoamyl alcohol; MxMglA, *M*. *xanthus* MglA; MxMglB, *M*. *xanthus* MglB; MxMglB^ΔCt^, MxMglB with Ct-helix truncated; nG, neonGreen; YPF, yellow fluorescent protein.

To test whether the MxMglB Ct-helix is important for MxMglB activity in vivo, we expressed MxMglB and the MxMglB^ΔCt^ variant under the control of *mglB* promoter at the *Mx8* phage attachment site in an *mglB* deletion mutant of *M*. *xanthus* (Δ*mglB*). MxMglB^ΔCt^ was stably expressed, although its expression level appeared slightly decreased compared to MxMglB (it is also possible that this apparent decrease is because the Ct-helix is an important epitope for the MxMglB antibody; S3A Fig, S3B Fig). MxMglB^ΔCt^ must nevertheless carry biological activity because it induced a drastic motility phenotype and localized at the poles (see below). MglA was expressed to comparable levels in strains complemented by MxMglB or MxMglB^ΔCt^ (S3A Fig, S3B Fig). The level of MglA is slightly reduced in a Δ*mglB* mutant because of a weak polar effect but this reduction did not create detectable phenotype [7]. The strain *mglB*^*+*^ complemented motility on an agar surface, while *mglB*^Δ*Ct+*^ led to profoundly defective motility in colony plate assays (Fig 5A). The motility phenotype was in fact more pronounced than for the Δ*mglB* mutant, suggesting that expression of MxMglB^ΔCt^ deeply perturbs the function of MglA. However, single cells were motile, and the defect could be linked, at least partially, to aberrant cell reversals (Fig 5B, S3C Fig, S3D Fig). In Δ*mglB* mutant cells, the polarity axis is disrupted and therefore the reversal distribution does not change depending on environmental conditions, contrarily to wild-type (WT) cells in which the reversals depend on the Frz-signaling state [7]. This can be shown in cells in which Frz signaling can be induced by addition of isoamyl alcohol (IAA), which increases the reversal frequency of *mglB*^+^ cells (Fig 5B) [7,22,23]. As expected, the Δ*mglB* mutant showed a reversal frequency distribution that did not change upon addition of IAA (Fig 5B). Remarkably, *mglB*^Δ*Ct+*^ cells also did not respond to the addition of IAA (Fig 5B), suggesting that the polarity axis and Frz-dependent regulations are deeply affected in absence of the MxMglB Ct-helix.

### MxMglA, MxMglB^ΔCt^, and RomR localize to both cell poles in mglB^ΔCt+^

To further explore if MxMglB^ΔCt^ perturbs cell polarity, we analyzed its localization in single cells of *M*. *xanthus* by expressing C-terminal neonGreen (nG) fused [24] to MxMglB and MxMglB^ΔCt^ in *mglB* deletion backgrounds. MglB-nG localized to the lagging cell pole and oscillated from pole-to-pole with kinetics comparable to previously described yellow fluorescent protein (YFP) and mCherry fusions (Fig 5C top, S3C Fig, S4A Fig) [4,5,11]. In contrast, MxMglB^ΔCt^-nG mostly localized symmetrically at both the poles and failed to oscillate (Fig 5C bottom, S3C Fig, S4A Fig). Thus, the Ct-helix is required for ensuring a unipolar localization of MxMglB.

We next tested how the bipolar localization of MxMglB^ΔCt^ affected the other polarity proteins, MxMglA and RomR, by expressing MxMglA-YFP or RomR-GFP, respectively, in the *mglB*^Δ*Ct+*^ strain. Remarkably, both MxMglA-YFP and RomR-GFP were bipolar and static (Fig 5D, Fig 5E [top], S4B Fig, S4C Fig), similar to the localization pattern observed in the Δ*mglB* deletion mutant [12,13]. Thus, in the presence of MxMglB^ΔCt^, the polarity axis is disrupted, exactly as observed when MxMglB is deleted. Most strikingly, all 3 proteins MxMglA, RomR, and MxMglB^ΔCt^ co-localize at the poles.

### Allosteric regulation in MglA homologues and other Ras-like GTPases

The regulation of MxMglA GTPase activity and MxMglAB interaction by MxMglB Ct-helix prompted us to explore whether analogous mechanisms exist among other prokaryotic and eukaryotic small Ras-like GTPases.

**Fig 6:**
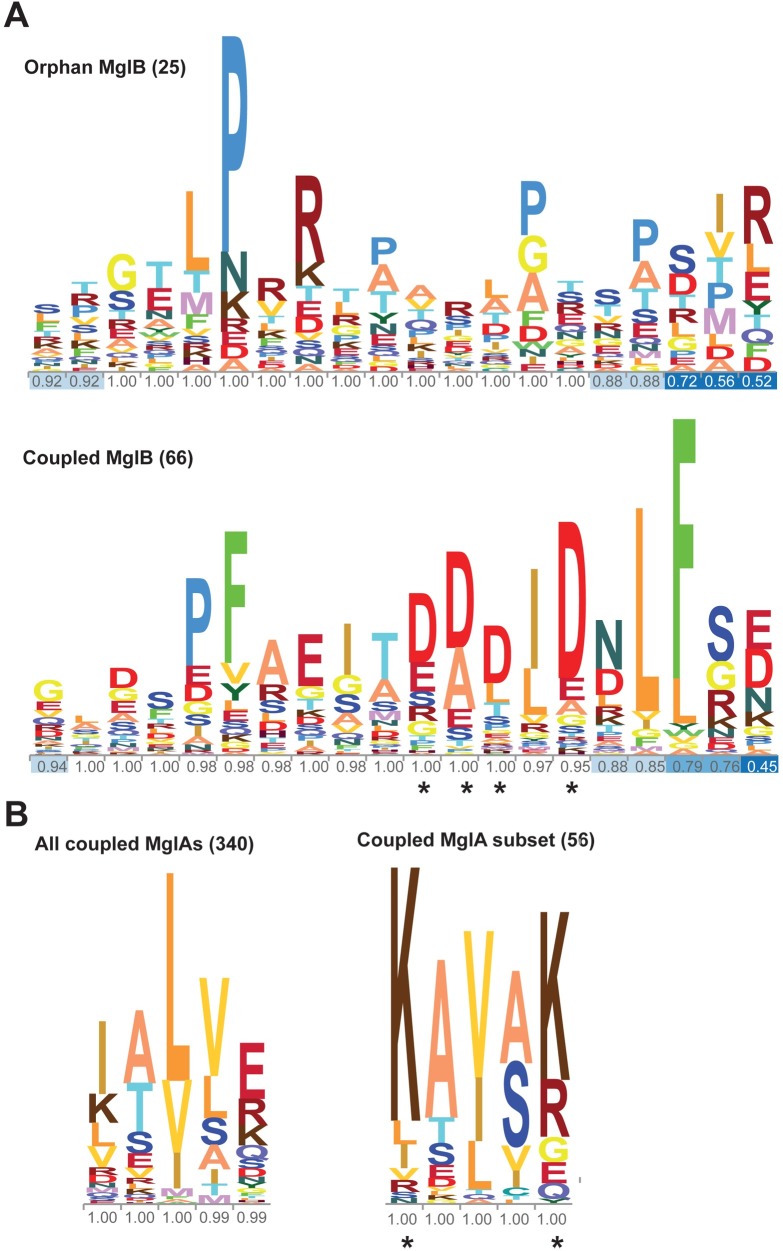
Conservation of sequence features of the allosteric pocket on MglA and MglB Ct-helix. (A) Comparison of residue conservation in C-terminal extension of coupled versus orphan MglB sequences. The aspartate residues implicated in interaction with the allosteric pocket on MglA are highlighted by “*” in the sequence conservation logo. The fractional occupancy of amino acids in the sequence alignment is shown for each amino acid position. The numbers within brackets denote the number of sequences in the alignment. The residues are colored in 20 different shades. (B) Comparison of residue conservation in the α_5_ helix of MglA sequences coupled with MglB sequences possessing a negatively charged C-terminal extension versus that of all coupled MglA sequences. The lysine residues that potentially interact with the Ct-helix of MglB are highlighted by “*” in the sequence conservation logo. The fractional occupancy of amino acids in the sequence alignment is shown for each amino acid position. The numbers within brackets denote the number of sequences in the alignment. Ct-helix, C-terminal helix; MglA, Mutual Gliding motility A; MglB, Mutual Gliding motility B.

Earlier, it has been shown that MglA and MglB, which exist in almost all phyla of eubacteria, can be categorized into 5 phylogenetically related groups [25]. Genes encoding MglB-like proteins are either associated with genes encoding MglA-like protein in the same operon, called coupled MglBs, or without an associated MglA gene (orphan MglBs) [25] (S3 Table). We analyzed these sequences and found that 66 coupled and 25 orphan MglBs possessed a C-terminal extension (more than 15 amino acids beyond the Rbl/LC7 fold; S3 Table). A total of 56 of the 66 coupled MglB sequences showed a predicted helical region containing 2 to 4 aspartates or glutamates (D/E rich), whereas the C-terminal extensions of the remaining MglB sequences contained proline-rich stretches with no predicted secondary structure (Fig 6A, S3 Table).

Out of these 56 coupled sequences, the α_5_ helix (which interacts with the Ct-helix) of 48 MglA sequences possessed positively charged residues (either lysines or arginines; Fig 6B). These positively charged residues were not conserved in other MglA sequences (Fig 6B). This suggested that the Ct-helix of MglB and its binding pocket on MglA have coevolved. Interestingly, we found MglB having the D/E rich Ct-helix to be present in all the 5 phylogenetic groups of MglA-like proteins, including a variety of bacteria and also in the archaea *Methanobacterium paludis* (S5 Fig). This indicated that the mechanism of allosteric regulation of the MglA GTPase by the MglB Ct-helix could be widespread in prokaryotic Ras-like GTPases and not limited to *Myxococcus*. Because the allosteric regulation by full-length MglB has not been characterized for any other prokaryotic MglA-like and MglB-like proteins, the mechanism proposed here would be relevant toward understanding their function.

## Discussion

### MxMglB Ct-helix facilitates nucleotide exchange allosterically

Our results revealed that the Ct-helix is essential for MxMglB to interact with MxMglA in the GDP-bound state. Deletion of the helix resulted in the formation of MxMglAB complex in the presence of only GTP but not GDP. This prompted us to identify the structural features that enable MxMglB to bind MxMglA–GDP. The β-screw movement (flipping of MxMglA β_2_ strand) appears to be essential for the interaction of MxMglB with MxMglA. This conformational change exposes residues that form a major part of MxMglAB interface.

**Fig 7:**
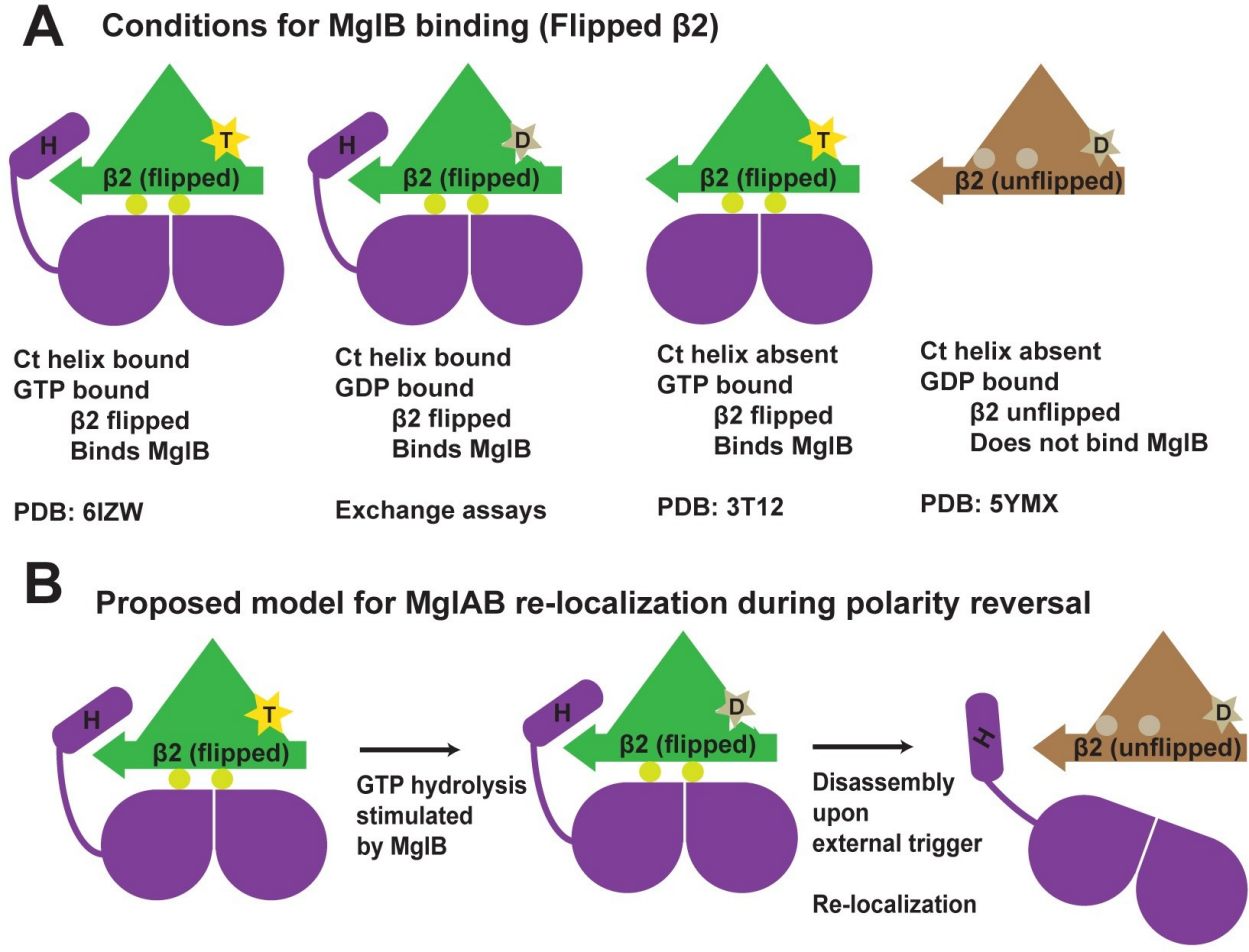
Ct-helix regulates MxMglAB interaction by binding to an allosteric pocket of the small Ras-like GTPase fold. (A) A schematic representation of the conditions that facilitate MxMglAB complex formation. PDB IDs 6IZW, 3T12, and 5YMX correspond to the crystal structures of MxMglAB–GTPγS, TtMglAB–GTPγS, and MxMglA–GDP complexes. (B) Proposed model for MxMglAB relocalization. Proteins that can bind in the allosteric pocket potentially compete out the MxMglB Ct-helix and dissociate the MxMglAB–GDP complex. In panels A and B, MxMglB dimer is shown in magenta, whereas MxMglA conformations with the flipped and unflipped states of β_2_ are shown in green and brown, respectively. A 6-pointed yellow star and a 5-pointed light brown star represent GTP (labeled as “T”) and GDP (labeled as “D”), respectively. The β_2_ strand is represented by an arrow. The residues that form the interface are schematically shown by 2 circles on bottom or top of the β_2_ strand representation in the flipped and unflipped states of the strand, respectively. Ct-helix of MxMglB is schematically shown by a cylinder and labeled as “H.” Ct-helix, C-terminal helix; MxMglA–GDP, MxMglA bound to guanosine 5’-diphosphate; MxMglA, *M*. *xanthus* MglA; MxMglAB, *M*. *xanthus* MglA and MglB complex; MglAB–GTPγS, MglA and MglB complex formed in the presence of guanosine 5’-O-[gamma-thio]triphosphate; PDB ID, Protein Data Bank identification.

An inspection of the MxMglAB–GTPγS structure revealed that the switch 1 loop and the binding pocket of the Ct-helix are at the N- and C-terminal ends of the β_2_ strand of MxMglA, respectively (Fig 2H). Hence, we propose that the flipped state of the β_2_ strand that favors MxMglB binding can be achieved in 2 ways—by interaction of either the γ phosphate of GTP with the switch 1 loop or the Ct-helix with the β_2_ to β_3_ loop (Fig 2H). The flipped state achieved by the Ct-helix interaction and the consequent stabilization by the Rbl/LC7 fold of MxMglB results in the formation of a complex with MxMglA–GDP. As a consequence of the flipped state of the β_2_ strand, MxMglA in the MxMglAB complex will potentially possess a nucleotide-binding pocket that favors binding of GTP over GDP, consistent with our observations from the nucleotide exchange assays (Fig 4). Thus, the proposed model provides an explanation for MxMglAB–GDP complex formation and a mechanistic insight into the allosteric action of Ct-helix for facilitating nucleotide exchange (Fig 7A).

Interestingly, like MglB, many eukaryotic GEFs such as DENN-1B domain, TRAPP-I, and Mon1-Ccz1, have the Rbl/LC7 fold [26]. Furthermore, we found that the intersubunit orientation in MglAB is conserved in the GTPase-GEF complexes of Rab35 with DENN-1B domain [27], Rab Ypt1p with TRAPP-I [28], and Ypt7 with Mon1-Ccz1 [29] (S6 Fig). However, unlike in MglAB complex, the mechanism of GEF action in these eukaryotic systems is based on a glutamate or aspartate residue interacting directly with switch 1 or switch 2 (S6A Fig, S6B Fig). These residues are present in extensions from the Rbl/LC7 fold or are contributed by other interacting partners that form the GEF complex [26].

### Mechanism of dual GAP and GEF action by MxMglB

We propose that MxMglB increases the rate of GTP hydrolysis by MxMglA through 2 complementary ways. Firstly, Rbl/LC7 domain of MxMglB stabilizes the flipped β_2_ strand interface, which orients the MxMglA catalytic residues favorably. Secondly, its Ct-helix, a flexible extension from the main Rbl/LC7 fold, retains the flipped state of the β_2_ strand post GTP hydrolysis and, consequently, stabilizes the MxMglAB–GDP complex. This interaction results in a nucleotide-binding pocket that accelerates release of GDP from the complex and favors GTP-binding. Thus, by facilitating GDP-to-GTP exchange MxMglB enhances the rate of GTP hydrolysis by MxMglA, increasing its potency as a GAP. Indeed, our in vitro results show that MxMglB^ΔCt^ exhibits reduced GAP activity, which we attribute to a defect in nucleotide exchange.

### Role of Ct-helix and exchange activity of MxMglB in the polarity oscillation cycle

In vivo, we propose that the MxMglB Ct-helix is critical for the balance between GAP and GEF activities at the lagging pole. In absence of the Ct-helix, all 3 proteins—MglA, MxMglB^ΔCt^, and RomR—localize to both cell poles. Thus, the MxMglB Ct-helix is absolutely required to enable the cascade of interactions that establish polarity and permit its switch. Co-localization of both MxMglA and MxMglB^ΔCt^ suggest that MxMglB^ΔCt^ does not support its GAP function in vivo, despite exhibiting only a reduction in GAP activity in vitro. In between reversals in wild-type cells, MxMglB and RomRX stably co-localize at the lagging pole [11,14,15]. MxMglA is efficiently excluded from the lagging pole, suggesting that the GAP activity of MxMglB predominates over the GEF activity of RomRX. Thus the MglB Ct-helix possibly overcomes the action of RomRX.

How could this function at the molecular level? Analysis of RomR revealed a stretch of negatively charged residues (Glu-rich) akin to that of MxMglB Ct-helix (S7A Fig). Thus, similar to MxMglB, the RomRX complex could facilitate GDP exchange from MxMglA by acting through the allosteric pocket on MxMglA. In this process, the C-terminal Glu-rich region of RomR might thus assist nucleotide exchange, whereas RomX could assist in stabilizing the flipped β_2_ strand of MxMglA. We tested this hypothesis by replacing the Ct-helix of MxMglB with the Ct-helix from RomR (S7A Fig, S7B Fig) and carrying out GTPase activity measurements in the presence of the chimeric MglB (MglB^Rhelix^). Indeed, we observed that the chimeric construct accelerated the GTPase activity of MglA similar to that of wild-type MxMglB (S7C Fig), supporting the hypothesis that the Ct-helix of RomR is capable of binding at the allosteric pocket of MxMglA. At the lagging pole, the MxMglB Ct-helix and the RomR Glu-rich helix might compete for binding at the MxMglA helix-binding pocket, which would displace RomRX from MxMglA. When the MglB Ct-helix is deleted, RomRX could therefore interact with MxMglA thus blocking MxMglB^ΔCt^ from exerting its GAP activity. This would allow RomRX to exert its GEF activity at the lagging pole too and stabilize the anchoring of MglA to both the poles.

Additional regulation of the Ct-helix must take place to determine the nucleotide specificity of MxMglB binding to MxMglA. Additional regulators in vivo will potentially determine the accessibility of the Ct-helix and thus the localization of MxMglA and MxMglB. At the pole, the exact molecular organization of the signaling complexes is not known. Other proteins that participate in the polarity switch and that are present at the lagging pole, for example, the MglB-like protein MglC, can potentially regulate the Ct-helix availability [30]. During the switch, action of FrzX^P^ could, for example, block the inhibitory effect of MglB on RomRX and thus initiate the switch. This possibility could be explored in the future. Further characterization of other polarity determining components and their interactions with MxMglAB are therefore essential to discriminate the GAP and GEF-active phases of MxMglB during the polarity reversal cycle.

Insights gained from the prokaryotic small Ras-like GTPase MxMglA and its interacting partner MxMglB highlight the dual role of MxMglB both as a GAP and a GEF. The novel mechanism of GEF action is based on an allosteric interaction with the small Ras-like GTPase. Based on sequence analysis, it appears to be a conserved mechanism among prokaryotic small Ras-like GTPases. The study also opens up a new avenue for design of compounds or factors that can modulate the enzyme activity by targeting the newly discovered allosteric binding pocket of the universal Ras-like fold.

## Methods and materials

### Protein expression and purification

#### Protein constructs, cloning, and overexpression

Genes corresponding to *mglA* and *mglB* were amplified from *M*. *xanthus* genomic DNA (obtained from DSMZ, Germany, catalogue number 16526) using suitable primers (S4 Table) and cloned into *pHis17* vector (obtained from Löwe lab, MRC LMB, Cambridge; refer Addgene plasmid #78201 for vector backbone) between the restriction enzyme sites *Nde*I (New England Biolabs Inc) and *BamH*I (New England Biolabs Inc). Single-point mutants of MxMglA and deletion constructs of MxMglB were generated using a site-directed mutagenesis strategy utilizing PCR-based methods followed by *Dpn*I (New England Biolabs Inc) digestion, transformation, and screening of positive clones. All clones were confirmed by sequencing. The list of clones and the primers used to generate them are summarized in S5 and S4 Tables. All chemicals were procured from Sigma Aldrich, unless otherwise mentioned.

#### Protein expression

Plasmids containing the gene of interest were transformed into suitable *Escherichia coli* strains, and culture was grown at 37°C and at 30°C post induction. *E*. *coli* strain BL21-DE3 was used for expressing MxMglA wild type and its mutants. The cultures were grown in LB media containing 50 μg/ml of ampicillin and induced with 0.5 mM IPTG at OD_600_ value of 0.8, whereas MxMglB and its mutants were expressed in the strain BL21-AI and induced with 0.02% L-arabinose (SRL Chemicals, India) at OD_600_ value of 0.6. Selenomethionine-labeled proteins for MxMglB^L156M^ and MxMglB^I148M^ have been expressed by the feedback inhibition method [31].

#### Purification of MxMglA(His_6_)

For purification, harvested cells were resuspended in the lysis buffer L (50 mM Tris, 200 mM NaCl [pH 8.0], and 10% glycerol) and then spun at 39,000*g* for 45 minutes at 4°C. Supernatant was loaded on to a 5-ml HisTrap (GE Lifesciences) column because the presence of hexahistidine tag at the C-terminus facilitated the binding of overexpressed protein of interest to the column. The column was equilibrated with binding buffer (Buffer A: 50 mM Tris [pH 8.0], 200 mM NaCl) prior to loading the supernatant and the bound protein was washed and eluted with a step gradient of 2%, 5%, 10%, 20%, 50%, and 100% of Buffer B (Buffer A containing 500 mM imidazole). The fractions containing the protein were pooled, concentrated, and loaded onto Superdex75, 10/300 (GE Lifesciences). The protein was eluted into 50 mM Tris (pH 8.0), 50 mM NaCl. Fractions containing the protein were pooled, concentrated, flash frozen, and stored at −80°C. MxMglA mutants, i.e., MglA^K^, MglA^L^, and MglA^Q^, were also purified using a similar protocol.

#### Purification of MxMglB

MxMglB(His_6_), MxMglB^ΔCt^(His_6_), and MxMglB^Rhelix^(His_6_) were purified using the same protocol as described for MxMglA(His_6_). For MxMglB constructs without histidine tag, i.e., MxMglB, MxMglB^3D^, and selenomethionine-labeled MxMglB^L156M^ and MxMglB^I148M^, ion-exchange chromatography was used to purify the proteins. First, the cells were resuspended in the lysis buffer (50 mM Tris [pH 8.0], 200 mM NaCl, 10% glycerol), and then spun at 39,000*g* for 45 minutes at 4°C. An ion-exchange column, QHP (5 ml, GE Lifesciences) was used to purify the protein. Buffers used for binding and elution were Buffer A (50 mM Tris [pH 8.0], 50 mM NaCl), and a linear gradient of Buffer A with Buffer B (50 mM Tris [pH 8.0], 1 M NaCl), respectively, ranging from 0% to 50% Buffer B over 20 column volumes. Fractions containing the protein of interest were pooled. This protein was dialyzed into Buffer A25 (50 mM Tris [pH 8.0], 25 mM NaCl) and again spun at 39,000*g* at 4°C, filtered, and loaded on MonoQ 10/100 (GE Lifesciences) to remove minor impurities present. Binding and elution buffers were the same as earlier. Fractions from the MonoQ run were checked on SDS-PAGE, and those containing protein of interest were concentrated and stored at −80°C.

### Crystallization and structure determination

#### MxMglA crystallization

About 500 conditions of commercially available screens (Molecular dimensions, Hampton Research) were screened using Mosquito crystallization robotic system, using drop sizes consisting of 100 nl of protein at a concentration of 10 mg/ml and 100 nl of crystallization cocktail, in 96-well sitting drop plates (MRC plate, SWISS-SCI). Initial hits were obtained in many of the conditions, and, further, it was reproduced and optimized to get well-diffracting crystals. For crystallization of MxMglA, protein was diluted to a final concentration of 10 mg/ml in A50 buffer (50 mM Tris [pH 8.0], 50 mM NaCl). Diffraction quality crystals were obtained using the following conditions in a drop ratio of 1:1 volumes for protein and crystallization condition: (i) 0.1 M sodium cacodylate (pH 6.5), 40% v/v 2,4-methyl pentane diol, and 5% PEG 8000; (ii) 0.1 M imidazole (pH 8.0), 30% w/v 2,4-methyl pentane diol, and 10% w/v PEG 4000; (iii) 0.1 M sodium citrate (pH 5.6) and 35% w/v tertiary-Butanol; 20% ethylene glycol was included as cryoprotectant in the parent condition during crystal freezing.

#### MxMglAB crystallization

MxMglA and MxMglB were mixed in a ratio of 1:1 (considering monomeric molecular weight of MglB) in A50 buffer containing 5 mM MgCl_2_ and 2 mM GTPγS (Sigma). The concentration of the proteins used to crystalize was 4 mg/ml of each. Screening for crystallization hits yielded a few conditions that were further optimized to get the desirable crystals. The crystals were obtained in 2 different conditions, namely, (i) PEG 4000 (8%) and ammonium sulphate (200 mM) and (ii) PEG 3350 (12%) and ammonium sulphate (200 mM). Diffraction quality crystals were obtained at a drop ratio of 1:1 volumes of protein to crystallization buffer. Crystallization conditions containing 20% PEG 400 were used as cryoprotectant during freezing of crystals. Crystals of MxMglA and selenomethionine-labeled MxMglB complex were obtained in the same crystallization conditions.

#### Structure solution and refinement

Diffraction data from the crystals were collected at the home source using Rigaku Micromax 007 X-ray generator, and higher resolution data and anomalous data were collected at the synchrotron sources at Diamond Light Source, Harwell, UK, and ESRF, Grenoble. Data reduction was performed using IMOSFLM [32] or XDS [33], and scaling using AIMLESS [34] in CCP4 package [35]. MxMglA and MxMglB structures were solved by molecular replacement using the TtMglAB structure (PDB code: 3T12). Molecular replacement was performed using PHASER [36] available in CCP4 package. Refinement was carried out using PHENIX package [37] and model building using *Coot* [38]. The refined structures have been deposited in the PDB with accession numbers 5YMX (MxMglA–GDP) and 6IZW (MxMglAB–GTPγS).

In order to confirm the registry of the amino acids belonging to the Ct-helix of MxMglB, selenomethionine-labeled protein for mutant constructs of MxMglB where Ile148^B^ and Leu156^B^, respectively, were mutated to methionines, were purified, and selenomethionine-labeled MxMglB was used for obtain MxMglAB crystals in the same crystallization condition. The anomalous data from the crystals were collected, and it was confirmed that the correct amino acids were modeled into the electron density. The anomalous signal from the methionines in these 2 mutants, respectively, confirmed the registry of the amino acids of the MxMglB Ct-helix (S1D and S1E Fig).

### Fluorescence anisotropy experiments

#### Binding studies using fluorescent anisotropy measurements

Binding of fluorescently-labeled nucleotides, *mant*-GDP (*m*-GDP) or *mant*-GppNHp (*m*-GNP) (Jena Bioscience) with the proteins were monitored by measuring the change in anisotropy [39]. The excitation and emission wavelengths used for monitoring the *mant*-labeled nucleotide fluorescence were 360 nm and 440 nm, respectively. The experiments were performed on Fluoromax-4 (Horiba), with a sample volume of 200 μl in a cuvette, and excitation and emission slit widths of 5 nm.

Nucleotide binding of MxMglA and its mutants were estimated by measuring the change in fluorescence anisotropy of the *mant*-nucleotide upon titration with increasing concentrations of the protein. Protein samples (MxMglA or its mutants) were titrated against a fixed concentration of *mant*-labeled nucleotide (100 nM). Each reading corresponds to 10 averaged single point anisotropy values. The initial value of anisotropy of the *mant-*labeled nucleotide was subtracted from all the values. GraphPad Prism was used to plot the values against the concentration of protein (concentration of MxMglA) and to fit the data to the binding equation for estimation of *K*_*D*_. Because it is known that MxMglA has a single binding site for nucleotides, equation for one site-specific binding, as given below, was used to fit the data points. The equation is y = B_max_ × x ÷ (*K_D_* + x), where y represents the bound fraction (fluorescence anisotropy based readout), B_max_ is the maximum value obtained, x is the concentration of MxMglA, and *K*_*D*_ is the binding constant.

The values were normalized by dividing each of them by the maximum value of anisotropy, B_max_. The normalized anisotropy values were plotted to obtain a binding curve. Because MxMglA was purified in the GDP-bound form, the measurements from this experiment are not absolute affinities of MxMglA (or its mutants) to the nucleotides but a reliable measure of exchange of the prebound GDP with *m*-GDP or *m*-GNP.

Similarly, anisotropy measurements for increasing amounts of MxMglB/MxMglB^ΔCt^ with nucleotide-bound MxMglA provided information about the binding affinity of MxMglB to nucleotide-bound MxMglA. A mix of 400 nM of *mant*-labeled nucleotide with 2 μM of MxMglA was titrated with increasing concentrations of MxMglB/MxMglB^ΔCt^. The same calculations as described above were performed to obtain the binding affinities (*K*_*D*_). In this case, the x-axis represents the concentration of MxMglB dimer. Because MxMglA binds to one dimer of MxMglB, equation for one site-specific binding was used to fit the data points.

Each data point is an average of at least 3 independent measurements, and the standard error is shown in which each data represent 3 to 5 repeats. Error bars represent standard error.

#### Nucleotide exchange assays

Intensity of fluorescence emission by *mant-*labeled nucleotide (GDP or GMPPNP [GNP]; Jena Bioscience) at 440 nm was monitored after the excitation at 360 nm. All the kinetic measurements for MxMglA, MxMglA with MxMglB, and their mutants were performed on Fluoromax-4 (Horiba), with a sample volume of 200 μl in a cuvette and excitation and emission slit widths of 2 nm. *m*-GDP/GNP (800 nM) was added with Buffer A50 (50 mM Tris, 50 mM NaCl, 5mM MgCl_2_ [pH 8.0]) in a quartz cuvette (10 × 2 mm path length), and the fluorescence intensity was monitored initially for 400 seconds. The protein, i.e., MxMglA or the mix of MxMglAB/MxMglAB^ΔCt^ (3 μM of MxMglA and 6 μM MxMglB/MxMglB^ΔCt^ monomer), was added in the cuvette at 400 seconds after stabilization of the signal from only *mant*-nucleotide. Consequently, the fluorescence was recorded for 1,400 seconds. The binding was estimated in terms of increase in fluorescence intensity. At 1,800 seconds, the *mant-*labeled nucleotide was competed out with excess of unlabeled nucleotide (GDP or GTP; 500 μM), resulting in a decrease of fluorescence intensity because of release of *mant*-labeled nucleotide from the protein. For plotting the relative intensities from the measurements, each value was divided by the average of first 200 readings (400 seconds). These accumulation and decay reactions were fitted to exponential binding equations as given below to estimate the *k*_*on*_ and *k*_*off*_ values.

P + N_1_ → PN_1_ + N_2_ → PN_2_ + N_1_, where P represents protein, N_1_ is the labeled nucleotide, N_2_ is the unlabeled nucleotide, and PN denotes the protein-nucleotide complex.

For estimation of *k*_*on*_, PN_t_ = PN_max_ (1 − e^−kt^).

For estimation of *k*_*off*_, PN_t_ = PN_max_ − PN_min_ (e^−kt^) + PN_min_

Here, PN_t_ represents the amount of the complex at time t, PN_max_ is the maximum amount of the complex upon association, and PN_min_ is the minimum amount of the complex after dissociation.

### GTP hydrolysis assay

#### NADH coupled assay

GTPase activity measurements were made using a coupled enzyme based assay in which the conversion of NADH to NAD^+^ is coupled to the utilization of GDP produced [20]. The GTPase or ATPase hydrolyses the nucleotide while pyruvate (PEP) kinase (PK) uses GDP (or ADP) and phosphoenol pyruvate to produce GTP (or ATP) and pyruvate. Lactate dehydrogenase (LDH) utilizes NADH and pyruvate to produce NAD^+^ and lactate. Because the utilization of NADH is equivalent to the GDP produced by the GTPase reaction, the actual read out is decrease in absorbance of NADH, which in turn estimates the GDP produced by the GTPase. The amount of NADH utilized in the reaction was measured by monitoring the NADH absorbance at 340 nm using a multiplate reader, Varioskan Flash (Thermo Scientific). A master mix was prepared in Buffer A50 containing PEP (1mM; Sigma), GTP (1mM; Jena Bioscience), NADH (600 μm; Sigma), and PK/LDH mix (approximately 25 U/ml; Sigma). All the components of NADH reaction were mixed in a total reaction volume of 200 μl and added in a 96-well plate. The reaction was initiated by addition of MxMglA or MxMglAB mix and their relevant mutant constructs. MxMglA and its mutants were used at 10 μM and MxMglB and its mutants at concentrations 20 μM and 200 μM (in a molar ratio of 1:2 and 1:10 considering monomeric molecular weight of MxMglB).

Readings were recorded at the interval of 20 seconds for 2 hours. The absorbance value of buffer containing GTP was subtracted from all the values. Also, the absorbance at the 0 time point for each reaction was subtracted from all readings of the reaction. The absorbance readings were represented as concentration of GDP released, by using a conversion factor based on the standard graph obtained by plotting the absorbance values for known concentrations of NADH. *k*_*cat*_ values were calculated and plotted using GraphPad Prism. Readings from 0 to 7,200 seconds were fitted to a line using linear regression, and the slope of the fitted line was calculated to obtain the amount of GDP released (in μM) per unit time. *k*_*cat*_ was calculated as the amount of GDP released per unit time per unit enzyme concentration. This value was used further to compare the enzyme activities among all the mutants. The significance was calculated using GraphPad Prism, using one-way ANOVA with Tukey test of 95% confidence interval, and significance was marked on the scatter plot.

#### Phosphate release measurements using malachite green assay

This assay was performed using MxMglA at a concentration of 10 μM, and MxMglB in molar ratio 1:2 (20 μM, considering monomeric molecular weight of MxMglB) with excess of GTP, i.e., 1 mM GTP in buffer A50 (50 mM Tris, 50 mM NaCl [pH 8.0]) containing 5 mM MgCl_2_. All the enzyme activity assays were performed at 30°C. Readings were taken at different time points, i.e., 0, 15, 30, 45, 60, 75, 90, 105, and 120 minutes. To stop the reaction, the sample was heated at 65°C and then malachite green reagent (prepared as described in [21]) was added. The absorbance was recorded at 630 nm after 20 minutes of incubation, using a plate reader (Varioskan, Thermo Scientific). Amount of Pi (inorganic phosphate) released in μM was calculated using a standard curve, obtained by mixing a set of known concentrations of NaH_2_PO_4_ with malachite green solution. The experiments were repeated multiple times, and the slope was calculated from every repeat. Readings from 0 to 120 minutes were fitted to a line using linear regression, and the slope of the fitted line was calculated to obtain the amount of Pi released (in μM) per unit time. *k*_*cat*_ was calculated as Pi released per μM enzyme concentration for each reaction. *k*_*cat*_ values were plotted, and significance was calculated on GraphPad Prism, using one-way ANOVA with Tukey test of 95% confidence interval, and significance was marked on the scatter plot.

### HPLC for bound nucleotide estimation

Purified MxMglA was diluted to 2 to 3 mg/ml concentration in buffer A50 and then heated at 65°C. The heated protein sample was spun at 21,000*g* for 10 minutes at 4°C. Then supernatant was filtered with 0.22 μm cellulose acetate filter (Corning), and the sample was loaded on the DNAPac 200 ion-exchange column (Thermo Fisher). Buffer A (2 mM Tris [pH 8.0]) was used as binding buffer. The runs were performed at a flow rate of 0.8 ml/min, with steps of 100% Buffer A for 3 minutes, followed by linear gradients of 0% to 30% Buffer B (2 mM Tris [pH 8.0], 1.25 M NaCl) for 10 minutes, and 30% to 100% B for 12 minutes. In S1B Fig, the peak profile obtained for 18 μM of MglA was compared with that of 50 μM GDP solution, with 35 μl of sample injection volume for both. The absorbance at 260 nm, indicative of the presence of GDP, was plotted against retention time.

### Thermal shift assay [40]

In a 25-μl reaction volume containing 2 μM of protein in buffer A50, SYPRO Orange dye (Sigma; available as a 5,000× concentration in DMSO) was added to a final concentration of 5×, in 96-well multiwell PCR plates. Biorad CFX96 Real-Time System was used to monitor the change in fluorescence of SYPRO Orange (Sigma-Aldrich). The mix was heated from 4°C to 90°C with an increment of 1.2°C per minute. Readings were recorded at intervals of 0.4°C. The melting temperature (T_m_) was calculated based on the differential of the fluorescence intensity versus temperature plot (dF/dT). This gave an estimate of the stability of the protein.

### In vivo experiments

#### *Myxococcus* strains, plasmids, growth conditions, and genetic constructs

Primers, plasmids, and strains used for this study are listed in S4 Table, S5 Table and S6 Table, respectively. In general, *M*. *xanthus* strains were grown at 32°C in CYE rich media as previously described by Bustamante et al. [23]. Plasmids were introduced in *M*. *xanthus* by electroporation. Complementation, expression of the fusion, and mutant proteins were obtained by ectopic integration of the genes of interest at the Mx8-phage attachment site [7] under the control of their own promoter in appropriate deletion backgrounds. *E*. *coli* cells were grown under standard laboratory conditions in Luria-Bertani broth supplemented with antibiotics, if necessary.

#### Construction of *Myxococcus* strains

Following an initial step of PCR amplification of the relevant gene fragments for complementation, the fragments were cloned into the pSWU19 vector by the one-step sequence- and ligation-independent cloning (SLIC) method reported previously by Jeong et al. [41]. Specific details of PCR amplification are given below, whereas S4 Table, S5 Table and S6 Table contain the list of primers, plasmids, and strains used.

pSWU19 *mglB*^ΔCt^, pSWU19 *mglB*^ΔCt^-neon green, and pSWU19 *mglB*-neon green for complementation of the *mglB* deletion were constructed by amplifying 200 bp upstream the *mglB* coding sequence (promoter) with the *mglB*^Δ*Ct*^ gene (417 bp of *mglB* gene), *mglB*^Δ*Ct*^ gene (417 bp) and the linker with neon green, and the *mglB* gene and the linker with neon green, respectively. We chose to use neonGreen because this fluorescent protein (FP) is exceptionally bright and strictly monomeric, preventing possible artifacts due to the formation of weak FP dimers [24].

#### Phenotypic assays

Soft agar motility was performed as previously described by Bustamante et al. [23]. In general, cells were grown up to an OD_600_ between 0.4 to 0.8 and concentrated at OD_600_ = 5. Then they were spotted (10 μl) on 0.5% agar in CYE (soft agar). Colonies were photographed after 48 hours.

#### Fluorescence imaging and fluorescence intensity measurements

Protocol followed is as reported previously by Guzzo et al. [11]. For phase-contrast and fluorescence microscopy, cells from exponentially growing cultures were concentrated to OD_600_ = 2 by centrifugation of 1 ml of culture and resuspended in the corresponding volume of TPM buffer (10 mM Tris-HCl [pH 7.6], 8 mM MgSO_4_, and 1 mM KH_2_PO_4_). Then a drop of 2 μl was deposited on a coverslip and covered with a 1.5% agar pad with TPM buffer. Microscopic analysis was performed using an automated and inverted epifluorescence microscope TE2000-E-Perfect Focus System (PFS; Nikon, France) with a 100×/1.4 DLL objective and a CoolSNAP HQ2 camera (Photometrics). Images were recorded and processed with Metamorph software (Molecular Devices). All fluorescence images were acquired with a minimal exposure time to minimize bleaching and phototoxicity effects.

#### Reversal scoring assay

Protocol followed is as reported previously by Guzzo et al. [11]. For this assay, cells were grown at 32°C in CYE medium. After overnight incubation, 1 ml of cells was centrifuged for 5 minutes at 7,000 rpm, and pellets were resuspended in TPM medium (10 mM Tris [pH 7.6], 8 mM MgSO_4_, 1 mM KH_2_PO_4_) to OD_600_ of 2 units. A total of 2 μl of cells were spotted on coverslip below a TPM 1.5% agar pad with addition of 0.075% IAA in melted agar before pouring the pad, allowing modulation of Frz-signaling intensity with IAA. IAA solutions were made in TPM buffer containing 1mM CaCl_2_. After 10 minutes of incubation at room temperature, time-lapse experiments were performed using an automated and inverted epifluorescence microscope TE2000-E- PFS (Nikon, France), with a 40x/0.75 DLL “Plan-Apochromat” objective and an ORCA-Flash4.0 LT PLUS Digital CMOS camera (#C11440-42U30 Hamamatsu Photonics). The microscope is equipped with a PFS that automatically maintains focus. Images were recorded with NIS-Eléments AR 4.60 software (Nikon).

#### Cell tracking

Image analysis was performed with a newly developed MicrobeJ/FIJI-based tracking procedure. Cells were detected on MicrobeJ by thresholding the phase-contrast images after stabilization. Cells were tracked using MicrobeJ on a minimum of 20 frames by calculating all object distances between 2 consecutive frames and selecting the nearest objects. The computed trajectories were systematically verified manually, and, when errors were encountered, the trajectories were removed. Reversals were scored when cells switched their direction of movement with a distance of reversal at least 0.3 μm long and the angle between the segments was less than 60°. The reversal frequency was calculated by dividing the number of reversals by the duration of the track to generate a reversal frequency per hour. Trajectories with a “distance mean” less than 0.3 μm were removed from the data set. For each strain, 2 movies at least per biological replicate acquired on 2 different days were analyzed and plotted on box plots with MicrobeJ.

#### Cluster counting

Image analysis was performed on FIJI with the plugin cell counter; fluorescence images were acquired with a 1-second exposure time for the strains expressing MxMglA-YFP and MxMglB-neonGreen (MglB-nG). A 200 ms exposure time was used for the strain expressing RomR-GFP. The fluorescent intensity profiles of the different strains were generated using MicrobeJ. Briefly, cells were straightened, and the pole of the cells with the strongest fluorescence signal was chosen to orient the cells. Cells with abnormal shapes, unusual fluorescence signals, or dividing were removed from the data set. For each strain, the fluorescence intensity profiles of hundreds of single cells were thus plotted on a same graph.

#### Western blot

Samples were grown at 32°C in CYE medium. When optical density at 600 nm (OD_600_) reached 0.4 to 1, a volume of culture equivalent to 1 ml was centrifuged for 5 minutes at 7,000 rpm. The pellet corresponding to whole cells was resuspended in 1X SDS-PAGE loading buffer containing β-mercaptoethanol for getting 10 OD_600_ units and heated for 10 minutes at 99°C. Proteins samples equivalent to 1 OD_600_ units were separated by SDS-PAGE. Electrophoresis was performed at 180 V for 50 minutes at room temperature using 10% SDS-polyacrylamide gel. For Western blotting, proteins were transferred from gels onto nitrocellulose membranes. The membranes were blocked 1 hour at room temperature in Tris-buffered saline (pH 7.6), 5% milk, 0.2% Tween 20 (for MglA) or in Tris-buffered saline (pH 7.6), 2% milk, 0.2% Tween 20 (for MglB) and incubated with primary antibodies directed against MxMglA (dilution at 1:5,000) or MxMglB (dilution at 1:2,500) in blocking buffer overnight at 4°C. Details of primary antibodies used are available in the work by Zhang et al. [7]. After 2 washings of 5 minutes with Tris-buffered saline (pH 7.6), 0.2% Tween, 20 membranes were incubated with Goat Anti-Rabbit IgG (H + L)-HRP Conjugate (#1706515 BioRad) in the respective blockings buffers. The peroxidase reaction was developed by chemiluminescence (SuperSignal West Pico Chemiluminescent Substrate #34080 Thermo Scientific) scanned and analyzed with ImageQuant LAS 4000 and TL analysis software (GE Healthcare life sciences).

### Sequence and structure analyses

The sequences were downloaded using the list given in the work by Wuichet and Søgaard-Andersen [25] from UniProt. The presence of the Ras-like GTPase and Rbl/LC7 domains, respectively, for the MglA and MglB sequences were confirmed using SMART (Simple Modular Architecture Research Tool) [42] analysis. The sequences were analyzed using JalView [43]. The MglB sequences were sorted according to their lengths to identify the sequences with C-terminal extension (longer than 15 amino acids beyond the last amino acid of the Rbl/LC7 fold). The coupled MglA sequences corresponding to MglB sequences with C-terminal extension were identified and analyzed for the presence of positively charged residues in α_5_ helix of MglA. Conservation-based logos were obtained from the sequence alignments using Skylign [44]. Phylogentic trees were generated using the default settings in Clustal Omega [45]. The tree data were analyzed using iTol [46]. The structures of small Ras-like GTPases and interactors containing Rbl/LC7 domain fold were identified using DALI server [47] by giving MxMglA and MxMglB structures as input.

## Supporting information

S1 Fig Confirmation of bound GDP and amino acid registry of MxMglB Ct-helix. (A) Electron density for the bound GDP (composite omit map F_0_-F_c_ shown at 2.5 σ). GDP is shown in ball-and stick representation. (B) HPLC profile confirming the presence of GDP (GDP from the protein extract is shown in black solid line whereas plot for the GDP standard in gray dotted line). The numerical data for the figure panel have been provided in the respective sheets in S1 Data. (C) Superposition of TtMglAB (gray; PDB 3T12) and MxMglAB structures (MxMglA in green, MxMglB in magenta). Cα traces of the 2 structures are shown. (D and E) Anomalous signal for registry confirmation of MxMglB Ct-helix sequence. Two different mutants of MxMglB were generated, I148M and L156M, and anomalous data collected from the crystals of MxMglAB–GTPγS complex, produced using selenomethionine-labeled MxMglB. Panel D represents anomalous map (orange mesh shown at 5σ) and crystal structure of MxMglAB–GTPγS complex determined with MxMglB^I148M^ mutant, whereas panel E represents the anomalous map calculated for MxMglB^L156M^ (orange mesh shown at 4σ), shown on superposed crystal structure of MxMglB^I148M^ mutant. Leu156^B^ and the methionine residues (MSE for selenomethionine) are labeled. MxMglA (green) and MxMglB (2 shades of magenta for MglB_1_ and MglB_2_) are shown as Cα traces, with the methionine side chains and Leu156^B^ shown in stick representation. (F) Sequence alignment of full-length MglB from *M*. *xanthus* (MxMglB) and *T*. *thermophilus* (TtMglB) and the truncated sequence of TtMglB in the crystal structure (3T1S). The amino acids deleted in the MxMglB^ΔCt^ construct are highlighted on the MxMglB sequence within the black box. Ct-helix, C-terminal helix; GTPγS, guanosine 5’-O-[gamma-thio]triphosphate; HPLC, high pressure liquid chromatography; Mx, *M*. *xanthus;* MxMglA, *M*. *xanthus* MglA; MglAB, MglA and MglB complex; MxMglB, *M*. *xanthus* MglB; MxMglB^ΔCt^, MxMglB with Ct-helix truncated; PDB, Protein Data Bank; Tt, *Thermus thermophilus*.
(TIF)

S2 Fig Mutational analysis for elucidating the role of residues involved in catalysis and allostery. (A) GTPase activities of wild-type MxMglA only (green), MxMglB only (dotted blue line) and MxMglAB (dark purple, MglAB 1:2; and light purple, MglAB 1:10). Ratio variations of 1:2 and 1:10 for MxMglB are shown in dotted and solid lines, respectively. The release of GDP was estimated using NADH-based enzyme-coupled assay. The lines represent the average of multiple repeats (at least 3), and the shaded zones represent standard error. The data shown for MxMglA and MxMglAB have been duplicated from Fig 3A for the sake of comparison. Reaction for MxMglAB 1:10 ratio has been repeated only twice and hence the line represents an average of 2 repeats without an error estimate. (B) Thermal shift assay for MxMglA (green), MxMglA^K^ (magenta), MxMglA^L^ (blue), MxMglA^Q^ (orange) demonstrating that the T_m_ for unfolding remains similar. (C) Fluorescence anisotropy measurements for MxMglB titrated against *m*-GDP–bound (dark orange) and *m*-GNP–bound (light orange) MxMglA^Q^, showing that binding affinities to MxMglB were not affected for the mutant (refer S2B Table for *K*_*D*_ values). (D) Fluorescence anisotropy measurements for MxMglA (green, circle), MxMglA^K^ (magenta, square), MxMglA^L^ (blue, triangle) titrated against *m*-GNP, demonstrating that the binding affinity for GNP remains similar (refer S2C Table for apparent *K*_*D*_ values). (E) Fluorescence anisotropy measurements for MxMglA (green, circle), MxMglA^K^ (magenta, square), MxMglA^L^ (blue, triangle) titrated against *m*-GDP, demonstrating that the binding affinity for GDP remains similar (refer S2C Table for apparent *K*_*D*_ values). (F) Fluorescence anisotropy measurements for MxMglB titrated against *m-*GNP- (light shade) or *m-*GDP–bound (dark shade) mutants of MxMglA, namely, MxMglA^K^ (magenta) and MxMglA^L^ (blue). (G) Kinetic measurements of increase in *m-*GDP fluorescence upon addition of MxMglA (green), mix of MxMglA and MxMglB in 1:2 ratio (dark purple), mix of MxMglA and MxMglB^ΔCt^ in 1:2 (magenta, solid line) and 1:10 (magenta, dotted line) ratios. *m*-GDP was added into the cuvette, and the fluorescence intensity monitored for 500 seconds, when either MxMglA or MxMglA+MxMglB or MxMglA+MxMglB^ΔCt^ were added. The time point of addition at 500 seconds is marked by a solid gray line labeled with “+ P.” The numerical data for all the figure panels have been provided in the respective sheets in S1 Data. *m*-GDP, 2’/3’-O-(N-Methyl-anthraniloyl)-guanosine-5’diphosphate; *m*-GNP, 2’/3’-O-(N-Methyl-anthraniloyl)-guanosine-5’-[(β,γ)-imido]triphosphate; MxMglA, *M*. *xanthus* MglA; MxMglA^K^, MxMglA K181A and K185A double mutant; MxMglA^L^, MxMglA L64A and I67A double mutant; MxMglA^Q^, MxMglA Q82L mutant; MxMglAB, *M*. *xanthus* MglA and MglB; MxMglB, *M*. *xanthus* MglB; MxMglB^ΔCt^, MxMglB with Ct-helix truncation; T_m_, melting temperature.
(TIF)

S3 Fig In vivo experiments in *M*. *xanthus* demonstrate that the Ct-helix is essential for polarity oscillations. (A) Stability of MglB^ΔCt^ in vivo. Detection of MglB by Western blots in extracts from WT (DZ2), *mglB* deletion mutant and *mglB* deletion mutant complemented by *mglB*^Δ*Ct*^ (*mglB*^Δ*Ct*+^), or *mglB*^*+*^. (B) Stability of MxMglA in WT and *mglB* mutant complemented by *mglB*^Δ*Ct*^ or *mglB*. Detection of MglA by Western blots in extracts from WT (DZ2), *mglB* deletion mutant and *mglB* deletion mutant complemented by *mglB*^Δ*Ct*^ (*mglB*^Δ*Ct*+^), or *mglB*^*+*^. (C) MxMglB Ct-helix is required for MxMglB oscillations. Micrographs show representative single cell examples. Scale bar = 4 μm. The curve represents average intensity profiles of the leading cell pole for MxMglB-nG (blue) or MxMglB^ΔCt^-nG calculated for *n* cells and shown before and after the reversal event (marked by time = 0, dotted line). Note that MxMglB^ΔCt^-nG fluctuations are centered at 0.5, indicating that the protein remains largely bipolar across the reversal event. Deviation: Standard error of the mean. (D) MglB Ct-helix is required for MglA-YFP oscillations. Micrographs show representative single-cell examples. Scale bar = 4 μm. The curve represents average intensity profiles of the leading cell pole for MxMglA-YFP calculated for *n* cells and shown before and after the reversal event (marked by time = 0, dotted line). Note that MxMglA-YFP fluctuations are centered at 0.5 when MglB^ΔCt^ is expressed, indicating that the protein remains largely bipolar across the reversal event. Deviation: Standard error of the mean. Ct-helix, C-terminal helix; MglA, Mutual gliding motility A; MglB, Mutual gliding motility B; MglB^ΔCt^, MglB with Ct-helix truncated; MxMglA, *M*. *xanthus* MglA; MxMglB, *M*. *xanthus* MglB; nG, neonGreen; WT, wild type; YFP, yellow fluorescent protein.
(TIF)

S4 Fig Truncation of the Ct-helix of MxMglB results in bipolar localization of MxMglB^ΔCt^, MxMglA, and RomR. (A) Fluorescence intensity profiles for MxMglB^ΔCt^-nG localization from individual cells (*n* represents the number of cells analyzed) of Δ*mglB* strain complemented with MxMglB^ΔCt^-nG (top) and MxMglB-nG (bottom), respectively. The brightest pole was chosen as the lagging pole in cells expressing MxMglB-nG. (B) Fluorescence intensity profiles for MxMglA-YFP localization from individual cells (*n* represents the number of cells analyzed) in the background of Δ*mglB* strain complemented with MxMglB^ΔCt^ (top) and MxMglB (bottom), respectively. The brightest pole was chosen as the leading pole in cells expressing MxMglA-YFP. (C) Fluorescence intensity profiles for RomR-GFP localization from individual cells (*n* represents the number of cells analyzed) in the background of Δ*mglB* strain complemented with MxMglB^ΔCt^ (top) and MxMglB (bottom), respectively. The brightest pole was chosen as the lagging pole in cells expressing RomR-GFP. The numerical data for the figure panel have been provided in the respective sheets in S1 Data. Ct-helix, C-terminal helix; MxMglA, *M*. *xanthus* MglA; MxMglB, *M*. *xanthus* MglB; MxMglB^ΔCt^, MglB with Ct-helix truncated; nG, neonGreen;RomR, Required for Motility Response Regulator;YFP, yellow fluorescent protein.
(TIF)

S5 Fig Conservation of D/E rich Ct-helix in MglA-like proteins. A phylogenetic tree based on MglB sequences with a Ct-helix extension (longer than 15 residues beyond the RBl/LC7 domain) and a corresponding positively charged α_5_ helix. The bacterial phyla demonstrate that these belong to varied classes and are not restricted to *Myxococcus*. MxMglB (UniProt ID: Q1DB03) and TtMglB (UniProt ID: Q5SJ82) are highlighted in shaded boxes, whereas the sequences for which the positively charged residues are not present in the corresponding coupled MglA sequences are highlighted by a star. Ct-helix, C-terminal helix; D/E, aspartate or glutamate; MglA, Mutual gliding motility A; MglB, Mutual gliding motility B; MxMglB, *M*. *xanthus* MglB; RBl/LC7, Roadblock/LC7; TtMglB, *T*. *thermophilus* MglB.
(TIF)

S6 Fig Eukaryotic GTPases associated with GEFs of Roadblock/LC7 domain. (A) MxMglAB complex superposed on Rab GTPase and its GEF Mon1-Ccz1 complex (PDB ID: 5LDD). (B) MxMglAB complex superposed on Yptp1 GTPase and TRAPP1 complex (PDB ID: 3CUE). The GTPase domains are shown in shades of green (dark green for eukaryotic GTPases, and pale green for MxMglA), the Rbl/LC7 domains in shades of magenta (dark shades for eukaryotic proteins and light pink for MxMglB), and insertions to the Rbl/LC7 fold and other associated proteins in gray. The insertions to the Rbl/LC7 fold or associated protein loops that contribute to the GEF activity are highlighted in dark orange and boxed, whereas the Ct-helix of MglB is in light orange. Ct-helix, C-terminal helix; GEF, guanine nucleotide exchange factor; MglB, Mutual gliding motility B; MxMglB, *M*. *xanthus* MglB; *M*. *xanthus* MglB; PDB ID, Protein Data Bank identification; Rbl/LC7, Roadblock/LC7.
(TIF)

S7 Fig Comparison of MxMglB Ct-helix with RomR Glu-rich sequence. (A) Sequence alignment of RomR Glu-rich C-terminal sequence with MxMglB Ct-helix sequence. Negatively charged residues are shown in blue, hydrophobic residues in red, and positively charged residues in pink. The conservation of amino acids is marked according to ClustalO format [45]. “*,” “:,” and “.” denote identity, similarity, and conservation of polar residues. (B) Secondary structure prediction of C-terminal end of RomR highlighting that the C-terminal region has helical features (highlighted by pink cylinder). Only the helical region (residues 375 to 417) following a long unstructured stretch from 125 to 370 residues is shown. The figure was generated using PSIPRED [48]. (C) GTPase activities of wild-type MxMglA only (green) and in the presence of MxMglB (dark purple; MglAB), MxMglB^ΔCt^ (magenta; MglAB^ΔCt^), and MxMglB^Rhelix^ (orange; MglAB^Rhelix^). MxMglA and MxMglB variants were used in a ratio of 1:2 considering monomeric molecular weight of MxMglB. The release of GDP was estimated using NADH-based enzyme-coupled assay. The lines corresponding to MxMglAB and MxMglB^Rhelix^ are shown with shaded zones depicting the error represent the average of at least 3 repeats, whereas MxMglB^ΔCt^ and MxMglA are shown without highlighting error because the data in this figure panel represent only 2 repeats. The numerical data for the figure panel have been provided in the respective sheets in S1 Data. Ct-helix, C-terminal helix; Glu, glutamate; MglAB^ΔCt^, MglA and MglB with Ct-helix truncated; MglAB^Rhelix^, MglA and MglB with RomR helix instead of Ct-helix; MxMglA, *M*. *xanthus* MglA; MxMglB, *M*. *xanthus* MglB; MxMglB^ΔCt^, MglB with Ct-helix truncated; MxMglB^Rhelix^, MglB with RomR helix instead of Ct-helix; RomR, Required for Motility Response Regulator.
(TIF)

S1 Table Crystallographic data collection and refinement statistics. (XLSX)

S2 Table Summary of binding affinities estimated by fluorescence anisotropy measurements. (XLSX)

S3 Table Summary of prokaryotic MglA and MglB sequences analyzed with respect to the presence of the Ct-helix in MglB and an associated MglA. Ct-helix, C-terminal helix; MglA, Mutual gliding motility A; MglB, Mutual gliding motility B.
(XLSX)

S4 Table List of primers for generating the relevant constructs. (XLSX)

S5 Table List of constructs. (XLSX)

S6 Table *M*. *xanthus* strains used for study. (XLSX)

S1 Data Numerical data for all the graphs reported in the paper are given in S1_Data. The coordinates of the crystal structures reported in the manuscript have been deposited in the PDB and the accession numbers are 5YMX (MxMglA–GDP) and 6IZW (MxMglAB–GTPγS). GTPγS, guanosine 5’-O-[gamma-thio]triphosphate; MxMglA, *M*. *xanthus* MglA; PDB, Protein Data Bank.
(XLSX)

## Abbreviations

Ct-helix: C-terminal helix
FP: fluorescent protein
Frz: *frizzy*
FrzX^P^: phosphorylated FrzX
FrzZ^P^: phosphorylated FrzZ
GAP: GTPase activating protein
GEF: guanine nucleotide exchange factor
GDP: guanosine di-nucleotide
GppNHp: guanosine-5’-[(β,γ)-imido]triphosphate
GNP: guanosine-5’-[(β,γ)-imido]triphosphate
GTP: guanosine tri-nucleotide
GTPγS: guanosine 5’-O-[gamma-thio]triphosphate
HPLC: high pressure liquid chromatography
IAA: isoamyl alcohol
LDH: lactate dehydrogenase
mant: 2’/3’-O-(N-Methyl-anthraniloyl)
*mgl*: mutual gliding motility
MglA: Mutual gliding motility A
MglB: Mutual gliding motility B
MxMglA: *M*. *xanthus* MglA
MxMglB: *M*. *xanthus* MglB
nG: neonGreen
PCR: Polymerase Chain Reaction
PDB: Protein Data Bank
PFS: Perfect Focus System
Pi: inorganic phosphate
PK: pyruvate kinase
Rbl/LC7: Roadblock/LC7
RomR: Required for Motility Response Regulator
SLIC: sequence- and ligation-independent cloning
SMART: Simple Modular Architecture Research Tool
TtMglA: *T*. *thermophilus* MglA
TtMglB: *T*. *thermophilus* MglB
WT: wild type
YFP: yellow fluorescent protein
