# Supplementary figures and images for: Allosteric regulation of a prokaryotic small Ras-like GTPase contributes to cell polarity oscillations in bacterial motility
Source: PLoS Biol. 2019 Sep 27;17(9):e3000459. doi: 10.1371/journal.pbio.3000459 (PMC6785124; doi:10.1371/journal.pbio.3000459)

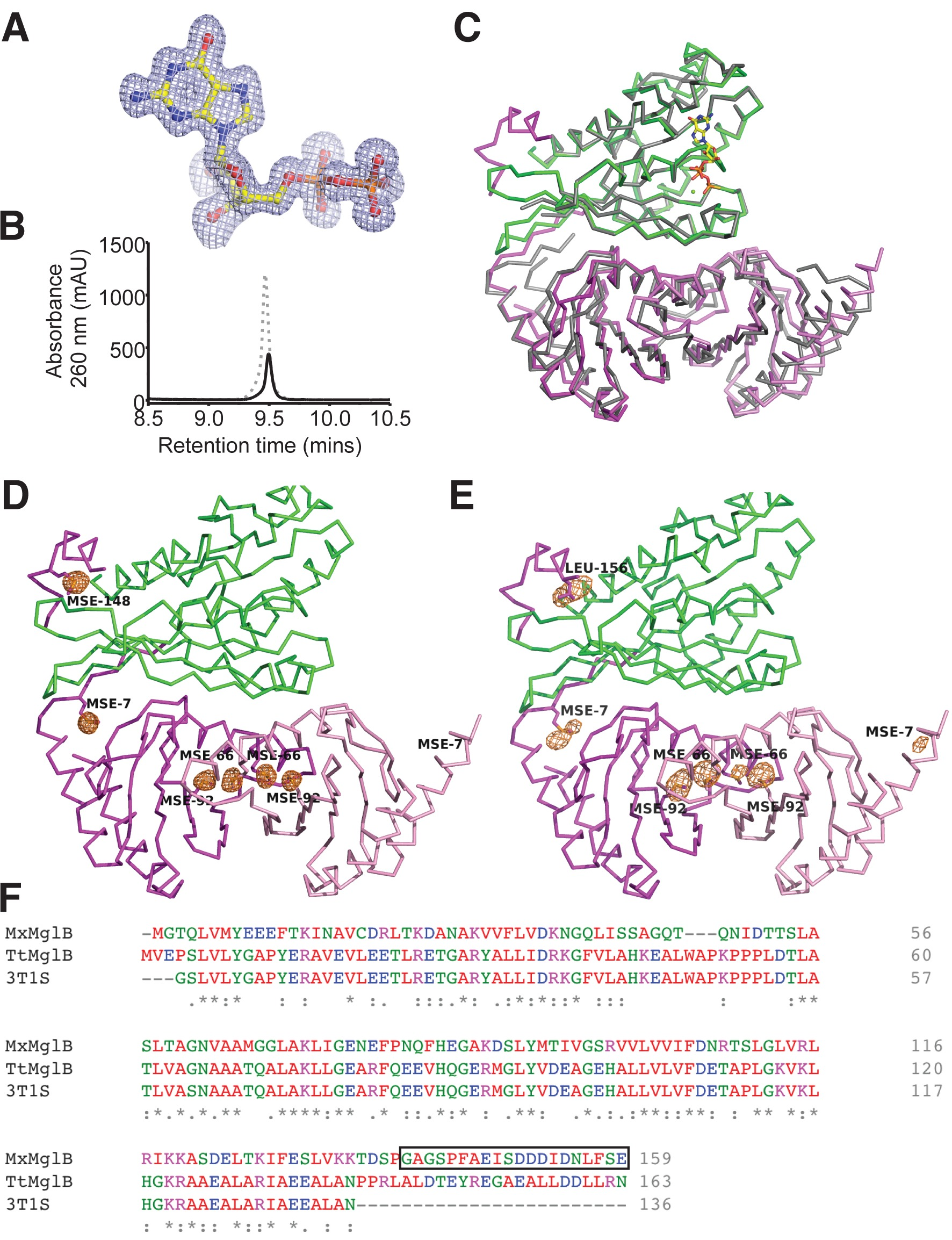

Supplement: S1 Fig — (A) Electron density for the bound GDP (composite omit map F0-Fc shown at 2.5 σ). GDP is shown in ball-and stick representation. (B) HPLC profile confirming the presence of GDP (GDP from the protein extract is shown in black solid line whereas plot for the GDP standard in gray dotted line). The numerical data for the figure panel have been provided in the respective sheets in S1 Data. (C) Superposition of TtMglAB (gray; PDB 3T12) and MxMglAB structures (MxMglA in green, MxMglB in magenta). Cα traces of the 2 structures are shown. (D and E) Anomalous signal for registry confirmation of MxMglB Ct-helix sequence. Two different mutants of MxMglB were generated, I148M and L156M, and anomalous data collected from the crystals of MxMglAB–GTPγS complex, produced using selenomethionine-labeled MxMglB. Panel D represents anomalous map (orange mesh shown at 5σ) and crystal structure of MxMglAB–GTPγS complex determined with MxMglBI148M mutant, whereas panel E represents the anomalous map calculated for MxMglBL156M (orange mesh shown at 4σ), shown on superposed crystal structure of MxMglBI148M mutant. Leu156B and the methionine residues (MSE for selenomethionine) are labeled. MxMglA (green) and MxMglB (2 shades of magenta for MglB1 and MglB2) are shown as Cα traces, with the methionine side chains and Leu156B shown in stick representation. (F) Sequence alignment of full-length MglB from M. xanthus (MxMglB) and T. thermophilus (TtMglB) and the truncated sequence of TtMglB in the crystal structure (3T1S). The amino acids deleted in the MxMglBΔCt construct are highlighted on the MxMglB sequence within the black box. Ct-helix, C-terminal helix; GTPγS, guanosine 5’-O-[gamma-thio]triphosphate; HPLC, high pressure liquid chromatography; Mx, M. xanthus; MxMglA, M. xanthus MglA; MglAB, MglA and MglB complex; MxMglB, M. xanthus MglB; MxMglBΔCt, MxMglB with Ct-helix truncated; PDB, Protein Data Bank; Tt, Thermus thermophilus. (TIF) [file pbio.3000459.s001.tif]

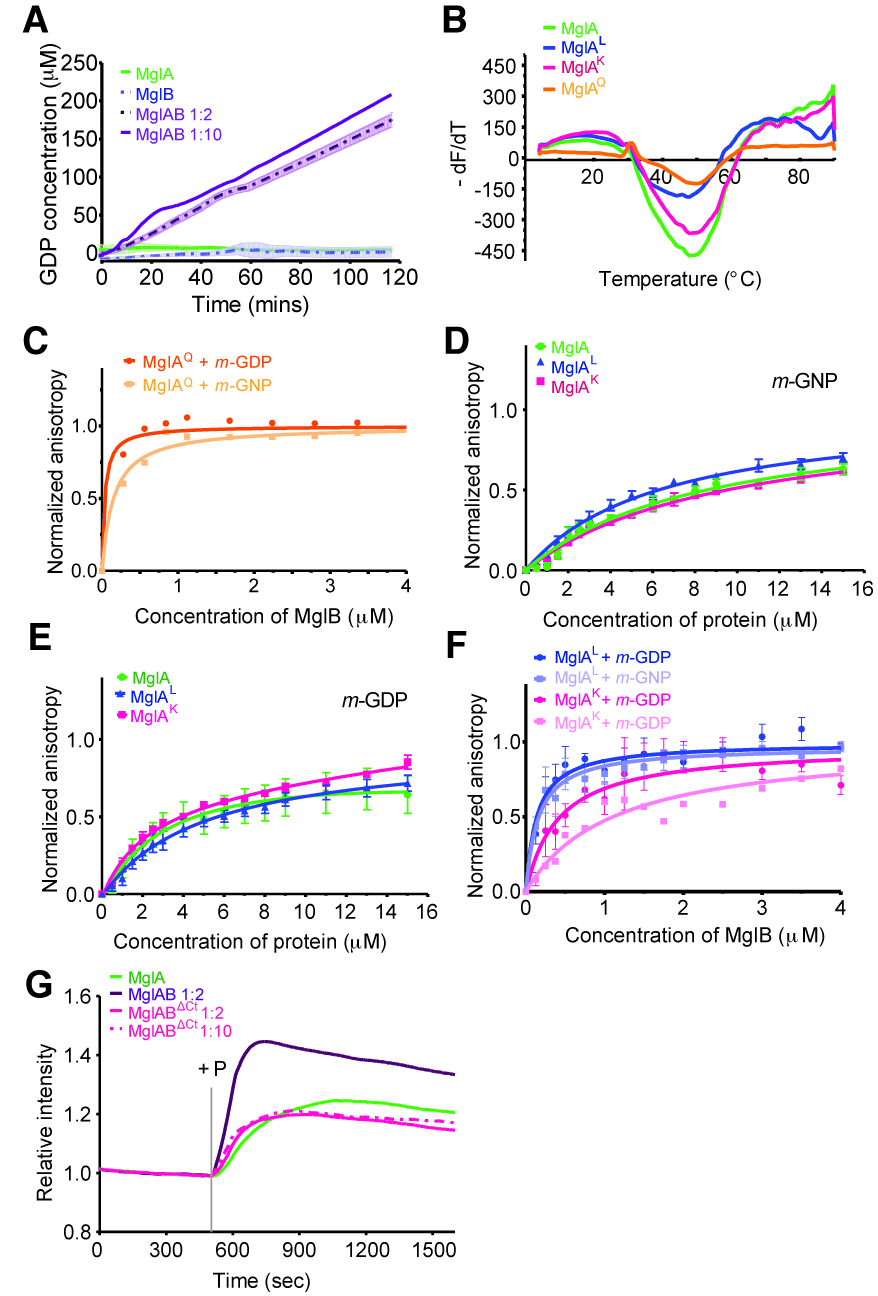

Supplement: S2 Fig — (A) GTPase activities of wild-type MxMglA only (green), MxMglB only (dotted blue line) and MxMglAB (dark purple, MglAB 1:2; and light purple, MglAB 1:10). Ratio variations of 1:2 and 1:10 for MxMglB are shown in dotted and solid lines, respectively. The release of GDP was estimated using NADH-based enzyme-coupled assay. The lines represent the average of multiple repeats (at least 3), and the shaded zones represent standard error. The data shown for MxMglA and MxMglAB have been duplicated from Fig 3A for the sake of comparison. Reaction for MxMglAB 1:10 ratio has been repeated only twice and hence the line represents an average of 2 repeats without an error estimate. (B) Thermal shift assay for MxMglA (green), MxMglAK (magenta), MxMglAL (blue), MxMglAQ (orange) demonstrating that the Tm for unfolding remains similar. (C) Fluorescence anisotropy measurements for MxMglB titrated against m-GDP–bound (dark orange) and m-GNP–bound (light orange) MxMglAQ, showing that binding affinities to MxMglB were not affected for the mutant (refer S2B Table for KD values). (D) Fluorescence anisotropy measurements for MxMglA (green, circle), MxMglAK (magenta, square), MxMglAL (blue, triangle) titrated against m-GNP, demonstrating that the binding affinity for GNP remains similar (refer S2C Table for apparent KD values). (E) Fluorescence anisotropy measurements for MxMglA (green, circle), MxMglAK (magenta, square), MxMglAL (blue, triangle) titrated against m-GDP, demonstrating that the binding affinity for GDP remains similar (refer S2C Table for apparent KD values). (F) Fluorescence anisotropy measurements for MxMglB titrated against m-GNP- (light shade) or m-GDP–bound (dark shade) mutants of MxMglA, namely, MxMglAK (magenta) and MxMglAL (blue). (G) Kinetic measurements of increase in m-GDP fluorescence upon addition of MxMglA (green), mix of MxMglA and MxMglB in 1:2 ratio (dark purple), mix of MxMglA and MxMglBΔCt in 1:2 (magenta, solid line) and 1:10 (magenta, dotted line) ratios. m [file pbio.3000459.s002.tif]

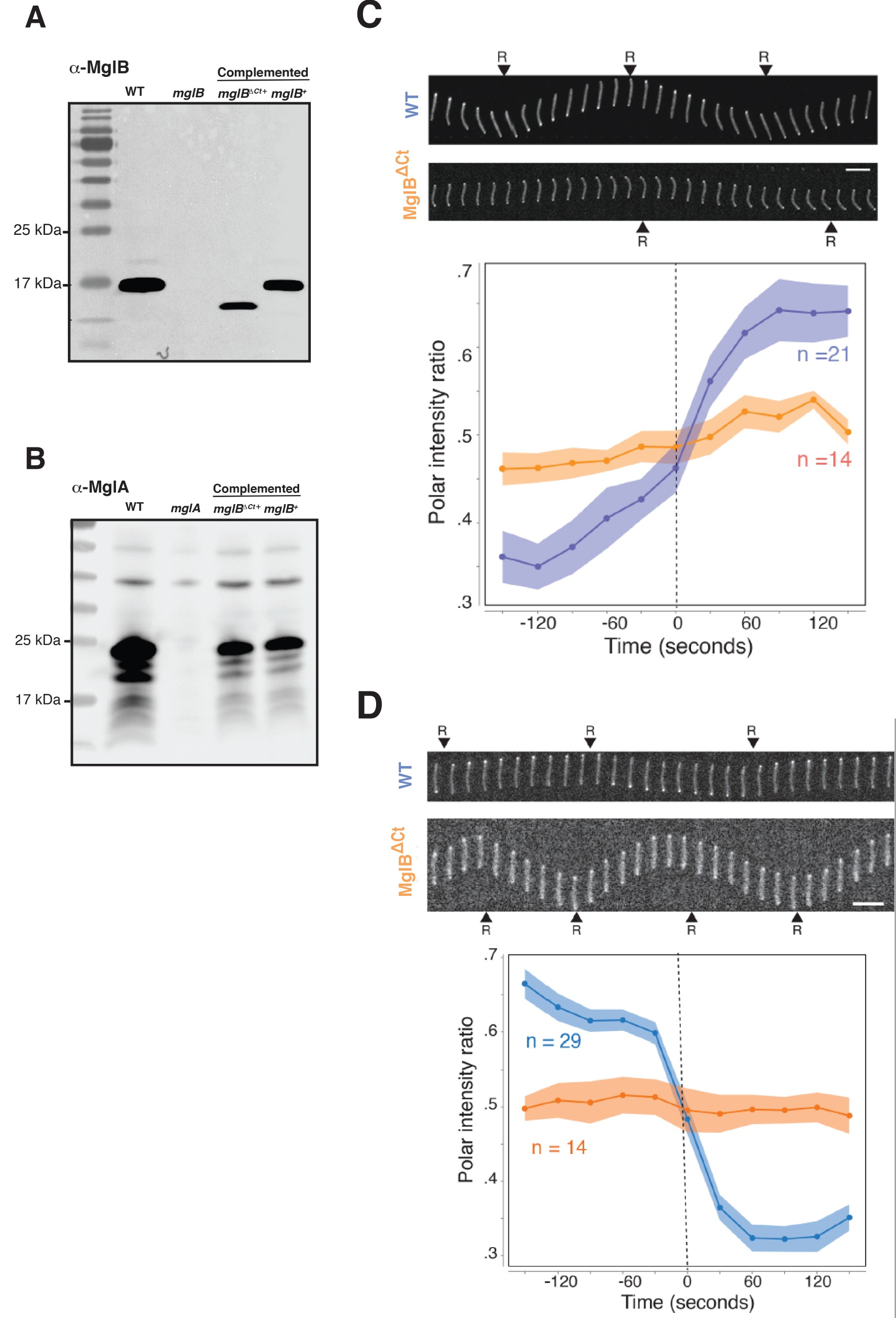

Supplement: S3 Fig — (A) Stability of MglBΔCt in vivo. Detection of MglB by Western blots in extracts from WT (DZ2), mglB deletion mutant and mglB deletion mutant complemented by mglBΔCt (mglBΔCt+), or mglB+. (B) Stability of MxMglA in WT and mglB mutant complemented by mglBΔCt or mglB. Detection of MglA by Western blots in extracts from WT (DZ2), mglB deletion mutant and mglB deletion mutant complemented by mglBΔCt (mglBΔCt+), or mglB+. (C) MxMglB Ct-helix is required for MxMglB oscillations. Micrographs show representative single cell examples. Scale bar = 4 μm. The curve represents average intensity profiles of the leading cell pole for MxMglB-nG (blue) or MxMglBΔCt-nG calculated for n cells and shown before and after the reversal event (marked by time = 0, dotted line). Note that MxMglBΔCt-nG fluctuations are centered at 0.5, indicating that the protein remains largely bipolar across the reversal event. Deviation: Standard error of the mean. (D) MglB Ct-helix is required for MglA-YFP oscillations. Micrographs show representative single-cell examples. Scale bar = 4 μm. The curve represents average intensity profiles of the leading cell pole for MxMglA-YFP calculated for n cells and shown before and after the reversal event (marked by time = 0, dotted line). Note that MxMglA-YFP fluctuations are centered at 0.5 when MglBΔCt is expressed, indicating that the protein remains largely bipolar across the reversal event. Deviation: Standard error of the mean. Ct-helix, C-terminal helix; MglA, Mutual gliding motility A; MglB, Mutual gliding motility B; MglBΔCt, MglB with Ct-helix truncated; MxMglA, M. xanthus MglA; MxMglB, M. xanthus MglB; nG, neonGreen; WT, wild type; YFP, yellow fluorescent protein. (TIF) [file pbio.3000459.s003.tif]

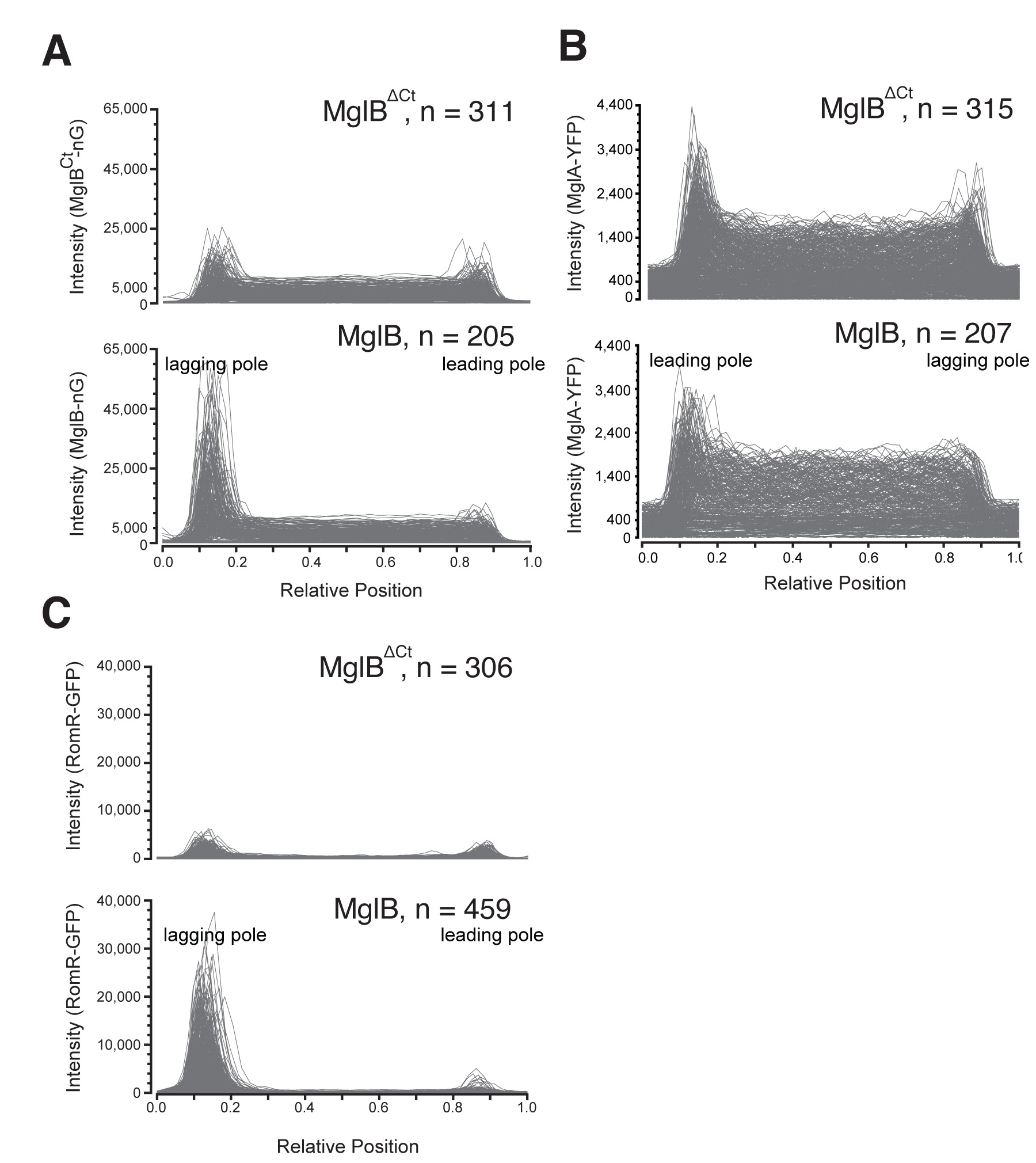

Supplement: S4 Fig — (A) Fluorescence intensity profiles for MxMglBΔCt-nG localization from individual cells (n represents the number of cells analyzed) of ΔmglB strain complemented with MxMglBΔCt-nG (top) and MxMglB-nG (bottom), respectively. The brightest pole was chosen as the lagging pole in cells expressing MxMglB-nG. (B) Fluorescence intensity profiles for MxMglA-YFP localization from individual cells (n represents the number of cells analyzed) in the background of ΔmglB strain complemented with MxMglBΔCt (top) and MxMglB (bottom), respectively. The brightest pole was chosen as the leading pole in cells expressing MxMglA-YFP. (C) Fluorescence intensity profiles for RomR-GFP localization from individual cells (n represents the number of cells analyzed) in the background of ΔmglB strain complemented with MxMglBΔCt (top) and MxMglB (bottom), respectively. The brightest pole was chosen as the lagging pole in cells expressing RomR-GFP. The numerical data for the figure panel have been provided in the respective sheets in S1 Data. Ct-helix, C-terminal helix; MxMglA, M. xanthus MglA; MxMglB, M. xanthus MglB; MxMglBΔCt, MglB with Ct-helix truncated; nG, neonGreen;RomR, Required for Motility Response Regulator;YFP, yellow fluorescent protein. (TIF) [file pbio.3000459.s004.tif]

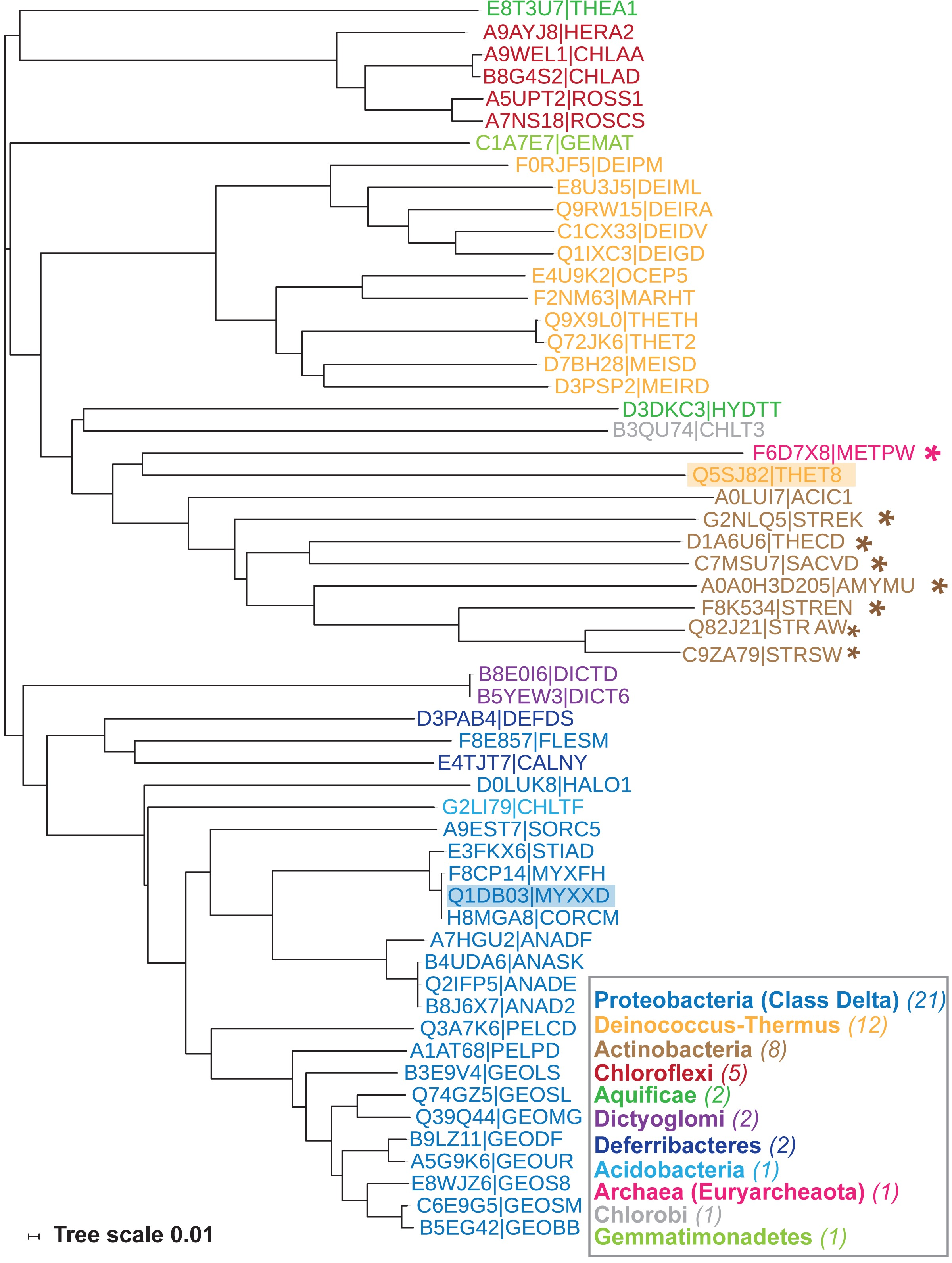

Supplement: S5 Fig — A phylogenetic tree based on MglB sequences with a Ct-helix extension (longer than 15 residues beyond the RBl/LC7 domain) and a corresponding positively charged α5 helix. The bacterial phyla demonstrate that these belong to varied classes and are not restricted to Myxococcus. MxMglB (UniProt ID: Q1DB03) and TtMglB (UniProt ID: Q5SJ82) are highlighted in shaded boxes, whereas the sequences for which the positively charged residues are not present in the corresponding coupled MglA sequences are highlighted by a star. Ct-helix, C-terminal helix; D/E, aspartate or glutamate; MglA, Mutual gliding motility A; MglB, Mutual gliding motility B; MxMglB, M. xanthus MglB; RBl/LC7, Roadblock/LC7; TtMglB, T. thermophilus MglB. (TIF) [file pbio.3000459.s005.tif]

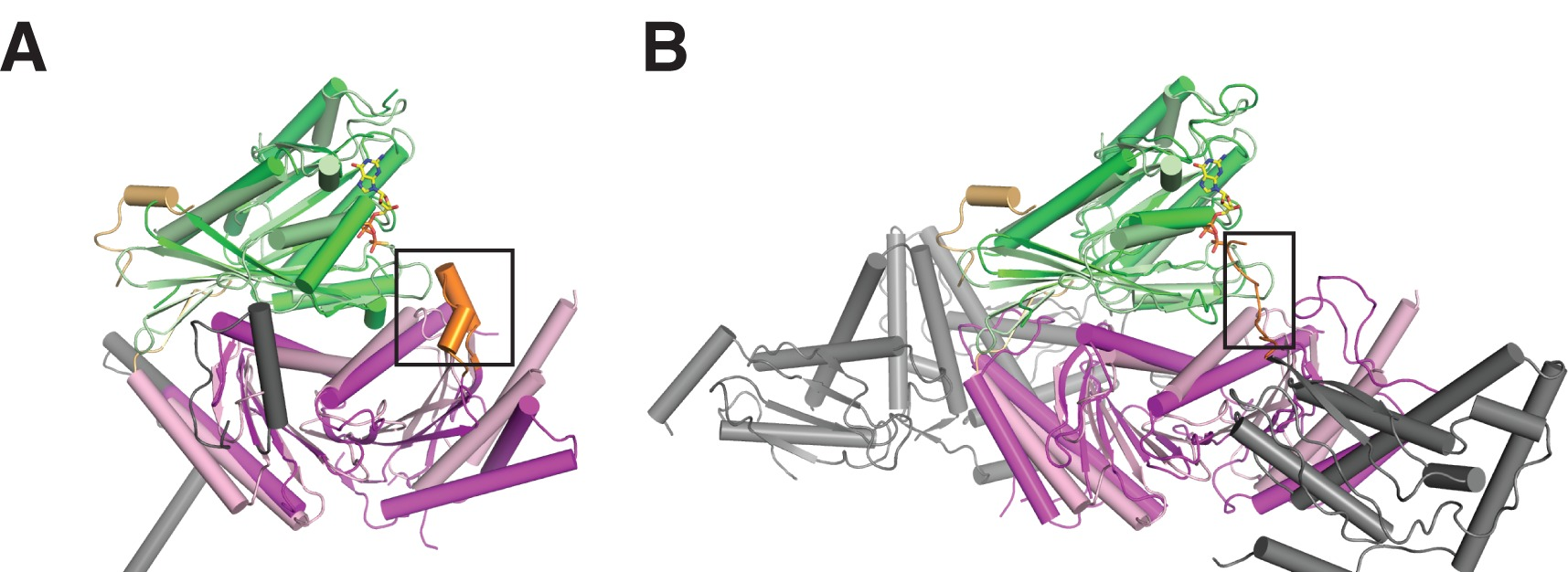

Supplement: S6 Fig — (A) MxMglAB complex superposed on Rab GTPase and its GEF Mon1-Ccz1 complex (PDB ID: 5LDD). (B) MxMglAB complex superposed on Yptp1 GTPase and TRAPP1 complex (PDB ID: 3CUE). The GTPase domains are shown in shades of green (dark green for eukaryotic GTPases, and pale green for MxMglA), the Rbl/LC7 domains in shades of magenta (dark shades for eukaryotic proteins and light pink for MxMglB), and insertions to the Rbl/LC7 fold and other associated proteins in gray. The insertions to the Rbl/LC7 fold or associated protein loops that contribute to the GEF activity are highlighted in dark orange and boxed, whereas the Ct-helix of MglB is in light orange. Ct-helix, C-terminal helix; GEF, guanine nucleotide exchange factor; MglB, Mutual gliding motility B; MxMglB, M. xanthus MglB; M. xanthus MglB; PDB ID, Protein Data Bank identification; Rbl/LC7, Roadblock/LC7. (TIF) [file pbio.3000459.s006.tif]

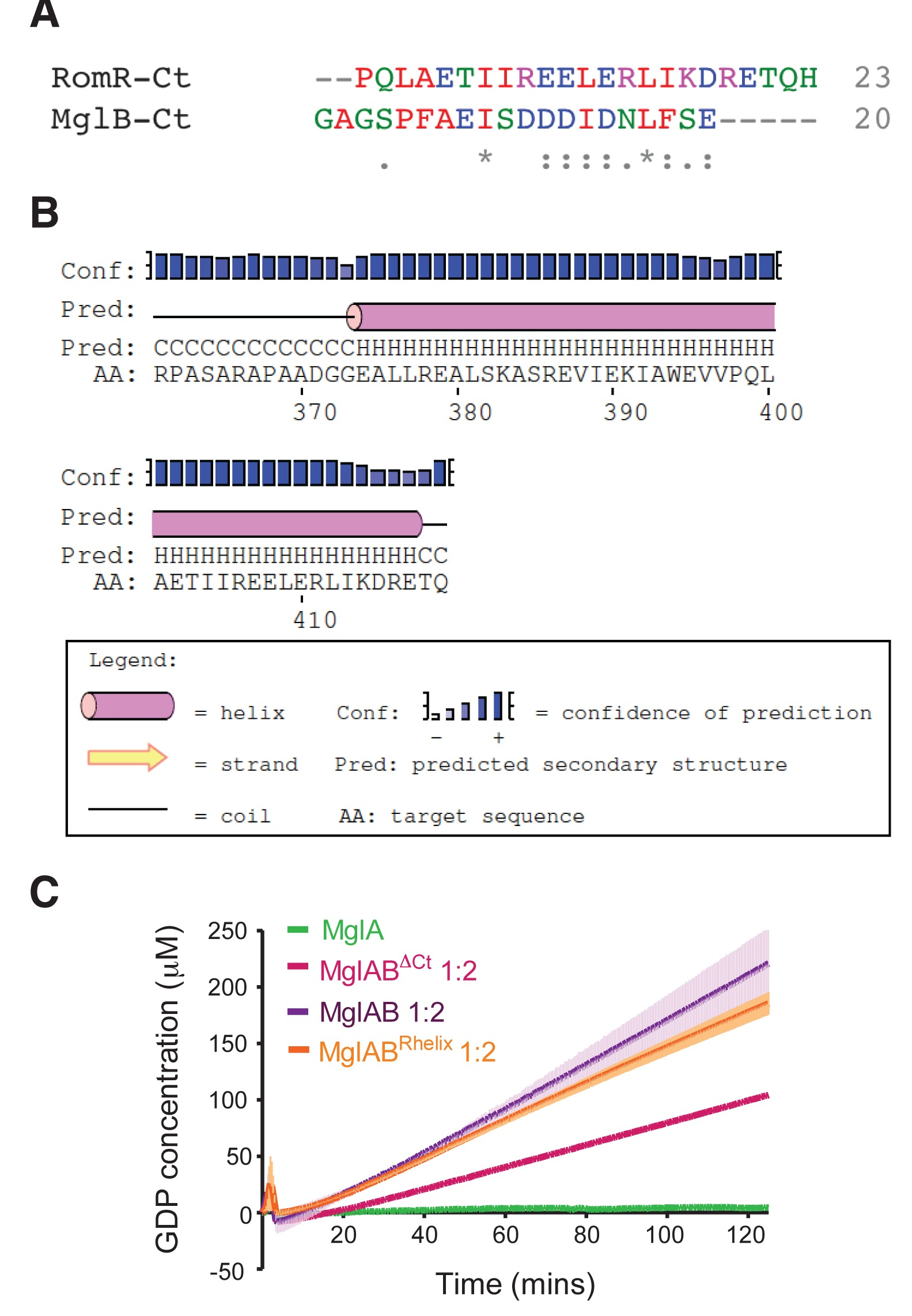

Supplement: S7 Fig — (A) Sequence alignment of RomR Glu-rich C-terminal sequence with MxMglB Ct-helix sequence. Negatively charged residues are shown in blue, hydrophobic residues in red, and positively charged residues in pink. The conservation of amino acids is marked according to ClustalO format [45]. “*,” “:,” and “.” denote identity, similarity, and conservation of polar residues. (B) Secondary structure prediction of C-terminal end of RomR highlighting that the C-terminal region has helical features (highlighted by pink cylinder). Only the helical region (residues 375 to 417) following a long unstructured stretch from 125 to 370 residues is shown. The figure was generated using PSIPRED [48]. (C) GTPase activities of wild-type MxMglA only (green) and in the presence of MxMglB (dark purple; MglAB), MxMglBΔCt (magenta; MglABΔCt), and MxMglBRhelix (orange; MglABRhelix). MxMglA and MxMglB variants were used in a ratio of 1:2 considering monomeric molecular weight of MxMglB. The release of GDP was estimated using NADH-based enzyme-coupled assay. The lines corresponding to MxMglAB and MxMglBRhelix are shown with shaded zones depicting the error represent the average of at least 3 repeats, whereas MxMglBΔCt and MxMglA are shown without highlighting error because the data in this figure panel represent only 2 repeats. The numerical data for the figure panel have been provided in the respective sheets in S1 Data. Ct-helix, C-terminal helix; Glu, glutamate; MglABΔCt, MglA and MglB with Ct-helix truncated; MglABRhelix, MglA and MglB with RomR helix instead of Ct-helix; MxMglA, M. xanthus MglA; MxMglB, M. xanthus MglB; MxMglBΔCt, MglB with Ct-helix truncated; MxMglBRhelix, MglB with RomR helix instead of Ct-helix; RomR, Required for Motility Response Regulator. (TIF) [file pbio.3000459.s007.tif]
